# Efficacy and safety of co‐crystal of tramadol‐celecoxib (CTC) in acute moderate‐to‐severe pain after abdominal hysterectomy: A randomized, double‐blind, phase 3 trial (STARDOM2)

**DOI:** 10.1002/ejp.2021

**Published:** 2022-09-24

**Authors:** Richard Langford, Adelaida Morte, Mariano Sust, Jesús Cebrecos, Anna Vaqué, Esther Ortiz, James Fettiplace, Shola Adeyemi, Grzegorz Raba, Liudmila But‐Husaim, Neus Gascón, Carlos Plata‐Salamán

**Affiliations:** ^1^ The London Clinic London UK; ^2^ ESTEVE Pharmaceuticals S.A. Barcelona Spain; ^3^ Mundipharma Research Limited Cambridge UK; ^4^ Medical College University of Rzeszów Rzeszów Poland; ^5^ Grodno City Clinical Hospital of Emergency Care Grodno Belarus; ^6^ GlaxoSmithKline Stevenage UK; ^7^ STATSXPERTS Consulting Limited Haverhill UK

## Abstract

**Background:**

STARDOM2 is a randomized, double‐blind, phase 3 trial evaluating the efficacy and safety of co‐crystal of tramadol‐celecoxib (CTC)—a first‐in‐class analgesic co‐crystal comprising racemic tramadol hydrochloride and celecoxib in a supramolecular network that modifies their pharmacokinetic properties—for the management of acute postoperative pain (NCT03062644; EudraCT:2016–000593‐38).

**Methods:**

Patients with moderate‐to‐severe pain following abdominal hysterectomy were randomized 2:2:2:2:2:1 to oral CTC 100 mg (*rac*‐tramadol hydrochloride 44 mg/celecoxib 56 mg) twice daily (BID); CTC 150 mg (66/84 mg) BID; CTC 200 mg (88/112 mg) BID; immediate‐release tramadol 100 mg four times daily (QID); celecoxib 100 mg BID; or placebo, for 5 days. The primary endpoint was the sum of pain intensity differences over 0–4 h (SPID_0–4_). Key secondary endpoints were rescue medication use within 4 h, 50% response rate at 4 h, and safety/tolerability.

**Results:**

Of 1355 patients enrolled, 1138 were randomized (full analysis set) and 1136 treated (safety analysis set). In the prespecified gatekeeping analysis of SPID_0–4_, CTC 200 mg was not superior to tramadol but showed non‐inferior efficacy (*p* < 0.001) that was sustained throughout the 120‐h period, despite a 5‐day cumulative tramadol administration of 880 mg with CTC 200 mg BID versus 2000 mg with tramadol 100 mg QID. Treatment‐emergent adverse events (TEAEs) and severe TEAEs were less common with CTC 200 mg versus tramadol. Treatment‐related TEAEs were 14.4% with CTC 200 mg and 23.6% with tramadol.

**Conclusions:**

Although the study did not meet its primary endpoint, CTC 200 mg showed a clinically relevant improvement in overall benefit/risk profile versus tramadol alone, with considerably lower cumulative opioid exposure.

**Significance:**

In the randomized, double‐blind, phase 3 STARDOM2 trial—in acute moderate‐to‐severe pain after abdominal hysterectomy—the novel co‐crystal of tramadol‐celecoxib (CTC) 200 mg BID was superior to placebo and non‐inferior to tramadol 100 mg QID. Although superiority to tramadol was not reached, CTC 200 mg BID exposed patients to lower cumulative opioid (tramadol) doses than tramadol (100 mg QID) alone, with fewer treatment‐emergent adverse events. CTC 200 mg thus has a clinically relevant improved benefit/risk profile compared with tramadol alone.

## 1 INTRODUCTION

There is an urgent need globally for new pain therapies that are both effective and better tolerated than current regimens (Gan, 2017; Gan et al., 2014; Sinatra, 2010). As part of this effort, multimodal analgesia is now recognized as the standard of care for acute postoperative pain that can arise from cutaneous, deep somatic, or visceral structures (Chou et al., 2016; Gan, 2017).

Co‐crystal of tramadol‐celecoxib (CTC), a first‐in‐class analgesic co‐crystal comprising racemic tramadol hydrochloride and celecoxib in a supramolecular network (Almansa et al., 2017), has been developed for the management of acute moderate‐to‐severe pain (Gascon et al., 2019). The recommended dosing regimen of 200 mg (*rac*‐tramadol hydrochloride 88 mg/celecoxib 112 mg) twice daily (BID) provides a relatively low daily dose of tramadol. By combining tramadol, a centrally acting weak μ‐opioid agonist and norepinephrine and serotonin reuptake inhibitor, with celecoxib, a non‐steroidal anti‐inflammatory drug (NSAID) that acts primarily via inhibition of cyclooxygenase‐2, CTC targets four different pathways for analgesia (Almansa et al., 2017; Gascon et al., 2019). In addition, the novel co‐crystal structure of CTC has been shown in preclinical and phase 1 studies to modify the physicochemical and pharmacokinetic (PK) properties of tramadol and celecoxib, optimizing these properties compared with their administration alone or in free combination. These differences include a lower maximum tramadol plasma concentration and prolonged time to reach this concentration, potentially reducing the frequency of adverse events (AEs), and a shortened time to maximum celecoxib plasma concentration, which may lead to a rapid onset of analgesia (Almansa et al., 2017; Cebrecos et al., 2021; Merlos et al., 2018; Port et al., 2019; Videla et al., 2018). CTC may therefore provide a means to effective pain relief, with reduced side effects and opioid consumption versus tramadol alone.

In a double‐blind, randomized, phase 2 trial in acute moderate‐to‐severe postoperative pain following oral surgery, CTC showed superior efficacy to placebo and tramadol, with comparable tolerability to tramadol (López‐Cedrún et al., 2018). This improved benefit/risk profile of CTC versus tramadol alone was confirmed in two phase 3 trials of acute moderate‐to‐severe pain: STARDOM1, in postoperative pain following oral surgery (López‐Cedrún et al., 2022), and SUSA‐301, in postoperative pain following bunionectomy with osteotomy (Viscusi et al., 2022).

Here, we report the findings of a third randomized, double‐blind, phase 3 trial (STARDOM2) that evaluated the efficacy and safety of CTC in acute moderate‐to‐severe pain following abdominal hysterectomy. The primary objective of this study, the first to examine CTC in a visceral pain model, was to demonstrate the efficacy of CTC by showing superiority over placebo, non‐inferiority to tramadol, and superiority over tramadol and celecoxib, with respect to the sum of pain intensity differences over the first 4 h (SPID_0–4_). A secondary objective was to compare the efficacy of CTC with tramadol and placebo by showing superiority for two key secondary endpoints: 50% responder rate at 4 h and use of rescue medication during the first 4 h. Other secondary objectives were to assess the safety and tolerability of CTC versus tramadol, celecoxib and placebo, and to evaluate efficacy with respect to a range of further endpoints.

## 2 METHODS

### 2.1 Study design and participants

STARDOM2 (NCT03062644; EudraCT: 2016–000593‐38) was a randomized, double‐blind, parallel‐group, placebo‐ and active comparator‐controlled phase 3 trial, conducted between 5 April 2017 and 29 June 2018, at 65 sites in seven countries (Belarus, Bulgaria, Hungary, Latvia, Poland, Russia, Spain).

Female patients, aged ≥18 years and in good general health, were eligible to participate if they were scheduled to undergo an elective total or subtotal abdominal hysterectomy with or without salpingo‐oophorectomy, under general anaesthesia via a Pfannenstiel incision, within 28 days of screening. Full inclusion and exclusion criteria and permitted and prohibited peri‐ and postoperative medications are listed in Supplementary Methods.

The study protocol was reviewed and approved by local ethics committees and all concerned competent authorities for each country and/or study site. Poland was the country of the principal investigator; the ethics committee for Poland was the Bioethics Committee at the Poznan University of Medical Sciences (resolution no. 17 February of 5 January 2017). All patients provided written informed consent. The study was conducted in accordance with Good Clinical Practice Guidelines and the Declaration of Helsinki.

### 2.2 Randomization and blinding

Before randomization, patients underwent a screening period of up to 28 days to confirm eligibility (Figure S1). After surgery—as soon as patients were calm and alert, and able to take fluids orally and walk 10 m without assistance—patients began pain assessments using an electronic diary. Patients rated their pain intensity on the pain intensity–visual analogue scale (PI‐VAS; 0 mm [no pain]–100 mm [worst imaginable pain]) every 30 min until a maximum of 30 h after surgery or until reaching a qualifying pain intensity (QPI; ≥45 to <70 mm [moderate]; ≥70 to ≤90 mm [severe]) for randomization.

Qualifying patients were randomized 2:2:2:2:2:1 to receive the following oral treatments for 5 days: CTC 100 mg (*rac*‐tramadol hydrochloride 44 mg/celecoxib 56 mg), 1 × 100‐mg capsule BID; CTC 150 mg (tramadol 66 mg/celecoxib 84 mg), 1 × 150‐mg capsule BID; CTC 200 mg (tramadol 88 mg/celecoxib 112 mg), 2 × 100‐mg capsules BID; immediate‐release tramadol 100 mg, 2 × 50‐mg capsules four times daily (QID); celecoxib, 1 × 100‐mg capsule BID; or placebo, 3 capsules QID. Randomization was performed using an automated system and stratified by QPI (moderate or severe). Patients and all personnel involved in study conduct or interpretation were blinded to treatment codes until completion. Treatments were over‐encapsulated to ensure identical appearance. Placebo capsules were included to ensure an equal total number of capsules per intake, and an equal number of intakes per day, across groups. Oral paracetamol was permitted as a rescue pain medication up to QID, to a maximum of 4000 mg/day. After the treatment period, patients were followed up for ≥7 days.

### 2.3 Assessments and endpoints

The primary efficacy endpoint was sum of pain intensity differences over 0–4 h (SPID_0–4_), with pain assessed using the PI‐VAS. SPID was defined as the weighted sum of pain intensity differences (baseline pain minus current pain).

Key secondary efficacy endpoints were the proportion of patients with a reduction in PI‐VAS ≥50% from 0 to 4 h (50% responder rate) and use of rescue medication within the first 4 h. Additional secondary efficacy endpoints included: SPID over 0–12, 0–24, 0–48, 0–72, 0–96 and 0–120 h; total pain relief (TOTPAR) over 0–4, 0–12, 0–24, 0–48, 0–72, 0–96 and 0–120 h; 30% responder rate at 4 h; time to perceptible and to meaningful pain relief; average dose of rescue medication per 24 h and time to first intake of rescue medication. Further details are provided in Supplementary Methods.

Safety was evaluated via monitoring of AEs, laboratory tests, vital signs, physical examination and electrocardiograms (ECGs). AEs were coded using the Medical Dictionary for Regulatory Activities, Version 19.0. Symptoms related to opioid use were assessed using the opioid‐related symptom distress scale (OR‐SDS) at 4, 24, 48, 72, 96 and 120 h, a 4‐point scale that evaluated distress dimensions (frequency, severity, bothersomeness) for 10 symptoms (fatigue, drowsiness, inability to concentrate, confusion, nausea, dizziness, constipation, itching, difficulty with urination, retching/vomiting).

### 2.4 Exploratory PK evaluation

Sparse blood samples were collected for exploratory PK analysis of tramadol, *O*‐desmethyltramadol (M1; a tramadol metabolite) and celecoxib levels. PK samples were collected at 2, 24, 50 and 72 h after first dosing. Plasma concentrations were determined via high‐performance liquid chromatography–tandem mass spectrometry analysis using an Agilent 1200 series pump (Sciex), and either an API4000 mass spectrometer detector (Sciex) or a Xevo TQ mass spectrometer (Waters Corporation), for celecoxib and tramadol/*O*‐desmethyltramadol respectively.

### 2.5 Statistical analysis

The full analysis set (FAS) comprised all randomized patients. The per‐protocol analysis set (PPAS) included all patients in the FAS with no major protocol deviations. The safety analysis set (SAS) included all patients who had received ≥1 dose of study drug. The PK analysis set included all patients with ≥1 PK measurement.

For the formal predefined analysis of the primary efficacy endpoint (SPID_0–4_) and key secondary endpoints (50% responder rate, and rescue medication use within the first 4 h), all three CTC doses were compared with placebo, tramadol and celecoxib using a parallel gatekeeping approach (hierarchical testing algorithm) to adjust for multiplicity (Bretz et al., 2009). The following four primary hypotheses were tested for each CTC dose in a hierarchical manner: superiority over placebo (FAS), non‐inferiority versus tramadol (PPAS), superiority over tramadol (FAS) and superiority over celecoxib (FAS). Testing was then performed for two secondary hypotheses in sequence: superiority over tramadol for 50% responder rate at 4 h, and superiority over tramadol for use of ≥1 dose of rescue medication during the first 4 h (FAS).

The significance level was split across the three CTC doses so that each test was performed at an initial alpha level of 1/3 of the usual one‐sided 2.5% level of significance (Bonferroni adjustment alpha = 0.0083). The testing sequence for the three CTC doses was applied in parallel.

Analysis of SPID_0–4_ was performed using analysis of covariance (ANCOVA). The last observation carried forward imputation method was used for missing PI‐VAS values. If rescue medication was taken, the most recent PI‐VAS value before the first intake of rescue medication was carried forward for all subsequent time points. The 50% responder rate and rescue medication use at 4 h were analysed using logistic regression.

Remaining secondary efficacy endpoints were summarized using exploratory descriptive analysis and analysed with appropriate statistical models. All *p* values were from two‐sided tests without adjustment for multiplicity. Descriptive statistics, including geometric means and coefficients of variation, were used for PK endpoints.

The sample size was calculated to enable detection of superiority of CTC over tramadol while also accounting for the need to first demonstrate CTC superiority over placebo and non‐inferiority to tramadol. A sample of 200 patients per active treatment arm was estimated to ensure a power of >90% for demonstration of CTC non‐inferiority to tramadol, if treatment differences were 20 mm∙h and assuming that 10%–20% of patients would not be included in the PPAS. A total of 1100 patients (200 per active arm, 100 for placebo) was determined to be sufficient to demonstrate CTC efficacy.

## 3 RESULTS

Of the 1355 patients enrolled, 1138 were randomized (FAS) and 1136 treated (SAS) (Figure S2). Of those randomized, 1066 (93.7%) completed the study, across all seven countries (Table S1). No single study centre accounted for more than 10% of patients who completed the study. Study completion rate was highest in the CTC 150‐mg group (197/206 [95.2%] patients completed) and lowest in the celecoxib group (193/205 [92.2%]). Of the 72 patients who discontinued the study, 46 (4.0%) withdrew consent, 19 (1.7%) discontinued due to AEs, 4 (0.4%) discontinued due to lack of efficacy, 2 (0.2%) were non‐compliant with the protocol, and 1 (0.1%) was lost to follow‐up. Overall, 850/1138 (74.7%) randomized patients were included in the PPAS.

Patient demographics and baseline characteristics were similar between treatment groups (Table 1). The mean (standard deviation) age of patients was 48.4 (6.98) years and approximately two‐thirds were overweight or obese, with a mean body mass index of 28.0 kg/m^2^. Mean QPI was approximately 64 mm and was moderate in 67.5% of patients and severe in 32.5%. Approximately two‐thirds of patients underwent a total abdominal hysterectomy and one‐third a subtotal abdominal hysterectomy. Surgery was bilateral in 895 patients, unilateral in 111, with location unreported in 132.

**TABLE 1:** Demographics and baseline characteristics (full analysis set)

Characteristic	CTC 100 mg (*n =* 207)	CTC 150 mg (*n =* 207)	CTC 200 mg (*n =* 208)	Tramadol 100 mg (*n =* 208)	Celecoxib 100 mg (*n =* 206)	Placebo (*n =* 102)
Age (years)						
Mean (SD)	48.2 (7.54)	48.2 (6.03)	48.5 (7.43)	48.7 (7.51)	48.1 (6.25)	48.7 (7.13)
Median (min, max)	48 (30–79)	48 (31–77)	48 (31–75)	48 (30–77)	48 (33–76)	48 (34–72)
Race, *n* (%)						
American Indian/Alaska native	–	–	–	–	1 (0.5)	–
Black/African American	–	–	1 (0.5)	1 (0.5)	–	–
White	207 (100)	207 (100)	207 (99.5)	206 (99.0)	204 (99.0)	102 (100)
Other	–	–	–	1 (0.5)	1 (0.5)	–
Weight (kg)						
Mean (SD)	74.9 (14.36)	75.1 (14.00)^a^	75.8 (14.5)	74.3 (13.3)	75.2 (14.7)^a^	75.4 (15.9)
Median (min, max)	74.0 (45.0, 115.0)	72.6 (44.0, 115.0)	73.9 (46.2, 127.5)	73.0 (50.0, 112.6)	73.6 (50.0, 130.0)	73.4 (51.8, 144.0)
Height (cm)						
Mean (SD)	164.4 (6.04)	164.8 (5.69)^a^	164.4 (5.89)	164.3 (5.91)	164.7 (6.25)^a^	164.3 (5.95)
Median (min, max)	164 (150, 180)	165 (150, 180)	165 (150, 180)	164 (143, 188)	164 (146, 187)	164 (149, 178)
BMI (kg/m^2^)						
Mean (SD)	28.0 (5.06)	27.9 (5.28)^a^	28.3 (5.38)	27.8 (5.09)	28.0 (5.22)^a^	28.1 (5.48)
Median (min, max)	27.3 (17.4, 42.8)	27.1 (16.4, 40.0)	27.6 (18.4, 45.4)	27.2 (18.3, 45.7)	27.2 (17.6, 51.4)	27.5 (19.3, 48.7)
Qualifying PI‐VAS score (mm)						
Mean (SD)	63.7 (10.39)	64.1 (11.40)	63.7 (11.57)	64.2 (10.98)	64.7 (11.08)^a^	64.2 (10.26)
Median (min, max)	63 (45, 87)	64 (45, 90)	63 (45, 93)	64 (45, 97)	64 (46, 94)	63.5 (45, 89)
Qualifying PI‐VAS score, *n* (%)						
Moderate (≥45–<70 mm)	141 (68.1)	139 (67.1)	140 (67.3)	142 (68.3)	137 (66.8)	69 (67.6)
Severe (≥70–≤90 mm)	66 (31.9)	68 (32.9)	68 (32.7)	66 (31.7)	68 (33.2)	33 (32.4)
Missing	–	–	–	–	1	–
Pre‐dose (0 h) PI‐VAS score (mm)						
Mean (SD)	65.1 (11.89)	64.8 (11.59)	64.3 (12.45)	64.4 (12.68)	65.4 (12.37)^a^	65.1 (11.34)
Median (min, max)	66 (9, 94)	64 (30, 90)	65 (29, 96)	63 (15, 90)	64 (27, 94)	65 (29, 88)
Pre‐dose (0 h) PI‐VAS score, *n* (%)						
Moderate (≥45–<70 mm)	131 (63.3)	131 (63.3)	137 (65.9)	140 (67.3)	132 (64.4)	66 (64.7)
Severe (≥70–≤90 mm)	76 (36.7)	76 (36.7)	71 (34.1)	68 (32.7)	73 (35.6)	36 (35.3)
Missing	–	–	–	–	1	–

Abbreviations: BMI, body mass index; CTC, co‐crystal of tramadol‐celecoxib; PI‐VAS, pain intensity–visual analogue scale; SD, standard deviation.

^a^ Two patients were randomized but not treated and did not provide baseline weight, height, or BMI. One did not provide a qualifying PI‐VAS score.

### 3.1 Efficacy

#### 3.1.1 Primary efficacy endpoint

**FIGURE 1:**
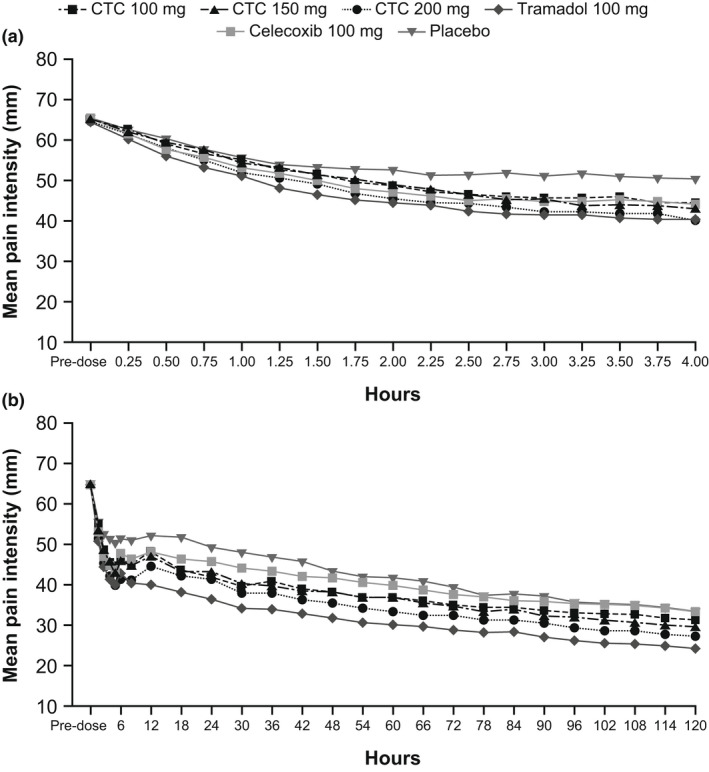
Mean pain intensity scores on the visual analogue scale (a) during the first 4 h post‐dose and (b) over 0–120 h (last observation carried forward, full analysis set). CTC, co‐crystal of tramadol‐celecoxib.

Mean PI‐VAS scores decreased rapidly in all groups over the first 2 h, then decreased at a slower rate over the remainder of the 120‐h assessment period (Figure 1). In the prespecified analysis of SPID_0–4_ (Table S2), CTC 200 mg demonstrated superiority over placebo (*p* < 0.05; FAS) and non‐inferiority to tramadol (*p* < 0.001; PPAS). No CTC doses demonstrated superiority over tramadol (or celecoxib) in the prespecified gatekeeping analysis (FAS); thus the primary endpoint was not met.

**FIGURE 2:**
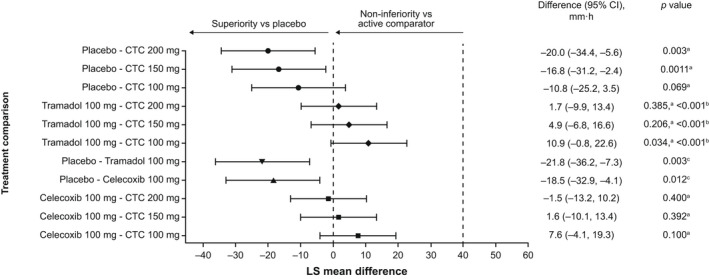
Sum of pain intensity differences over 0–4 h (last observation carried forward, full analysis set). Data were analysed using an analysis of covariance model with treatment and qualifying pain intensity at randomization (moderate or severe) as fixed effects, centre as a random effect, and pre‐dose (0 h) pain intensity as a covariate. ^a^ *p* value from one‐sided test of superiority for testing the null hypothesis that the difference of means is ≥0 mm∙h; ^b^ *p* value from one‐sided test of non‐inferiority for testing the null hypothesis that the differences of means is ≥40 mm∙h; ^c^ *p* value from two‐sided test of no difference for testing the null hypothesis that the differences of means is zero. CI, confidence interval; CTC, co‐crystal of tramadol‐celecoxib; LS, least‐squares.

In ANCOVA analyses of SPID_0–4_, CTC 150 mg (*p* = 0.011) and 200 mg (*p* = 0.003) were superior to placebo (FAS); the difference between CTC 100 mg and placebo did not reach significance (*p* = 0.069). All CTC doses showed non‐inferiority to tramadol (FAS; *p* < 0.001; Figure 2). Similar results were obtained for the PPAS. Results of subgroup analyses (QPI, age, type of surgery, country) were consistent with these findings (data not shown).

#### 3.1.2 Key secondary endpoints

**FIGURE 3:**
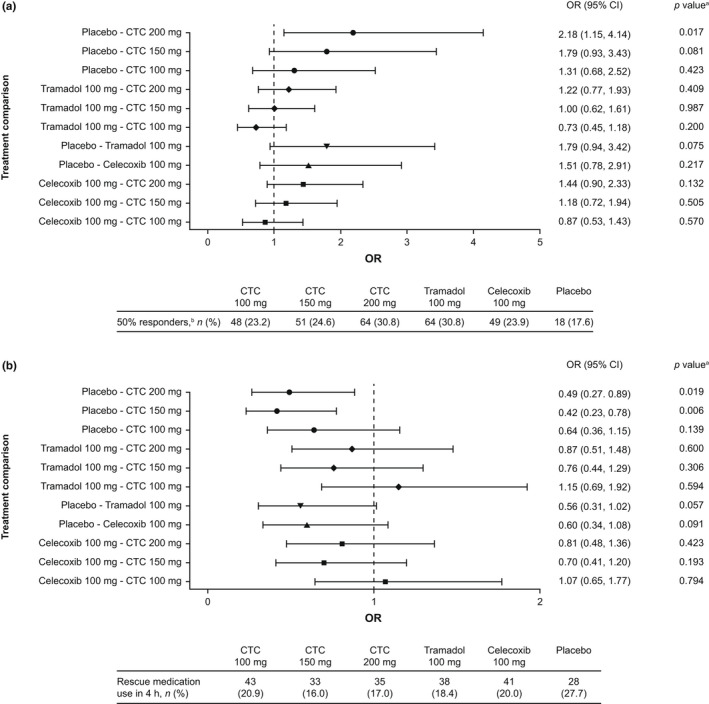
Summary of key secondary efficacy endpoints: (a) 50% responder rate at 4 h and (b) use of rescue medication during the first 4 h (full analysis set). Data were analysed using a logistic regression model with treatment and qualifying pain intensity at randomization (moderate or severe) as fixed effects, centre as a random effect, and pre‐dose (0 h) pain intensity as a covariate. ^a^ *p* value from two‐sided test of no difference for testing the null hypothesis that the OR = 1. ^b^50% reduction from baseline in pain intensity–visual analogue scale. CI, confidence interval; CTC, co‐crystal of tramadol‐celecoxib; OR, odds ratio.

In the FAS, the 50% responder rate at 4 h was 23.2% for CTC 100 mg, 24.6% for CTC 150 mg, 30.8% for CTC 200 mg, 30.8% for tramadol, 23.8% for celecoxib and 17.6% for placebo. In the gatekeeping analysis (FAS), CTC did not show superiority over tramadol, although in the logistic regression analyses, CTC 200 mg was superior to placebo (Figure 3a). Similar results were obtained in the PPAS.

In the FAS, 20.9% of patients used rescue medication in the first 4 h in the CTC 100‐mg group, 16.0% in the CTC 150‐mg group, 17.0% in the CTC 200‐mg group, 18.4% in the tramadol group, 20.0% in the celecoxib group and 27.7% in the placebo group. In the gatekeeping analysis (FAS), CTC did not show superiority to tramadol, although in logistic regression analyses, CTC 150 and 200 mg were both superior to placebo (Figure 3b). Similar results were observed in the PPAS.

#### 3.1.3 Additional secondary efficacy analyses

Significant differences were seen for CTC 150 and 200 mg versus placebo with respect to SPID over 0–12, 0–24, 0–48, 0–72, 0–96 and 0–120 h. CTC 200 mg showed the largest difference versus placebo at each time point (Table S3). Results were similar for TOTPAR over 0–4, 0–12, 0–24, 0–48, 0–72, 0–96 and 0–120 h. CTC generally did not differ significantly from tramadol or celecoxib at any dose or time point (Table S4).

The 30% responder rate at 4 h in the FAS was significantly different to placebo for CTC 150 mg (*p* = 0.049) and 200 mg (*p* = 0.018). CTC 200 mg differed significantly from celecoxib at most time points (Table S5). Trends were similar in the PPAS. The percentage of patients to achieve 30% response was highest in the CTC 200‐mg group (86.1%) and lowest in the placebo group (74.5%). A significant difference, using pairwise log‐rank tests, was observed between CTC 200 mg and placebo (*p* = 0.045). Similarly, the percentage of patients to achieve 50% response was highest with CTC 200 mg (78.4%) and lowest with placebo (67.6%). Significant differences were seen for CTC 200 mg versus placebo (*p* = 0.016) and for CTC 150 mg versus tramadol (*p* = 0.046). Time to perceptible pain relief did not differ significantly between treatment groups. Time to meaningful pain relief was significantly shorter for CTC 100 mg (*p* = 0.044) and 200 mg (*p* = 0.004) versus placebo.

Average daily rescue medication use in the FAS was significantly lower in the CTC 200‐mg than placebo (*p* = 0.017) group. Average daily rescue medication use was significantly lower for all CTC groups versus celecoxib (*p* < 0.05; Table S6). Similarly, pairwise log‐rank tests (FAS) showed that time to first intake of rescue medication was significantly longer for CTC 200 mg than placebo (*p* = 0.017), and for CTC 200 mg (*p* = 0.038), 150 mg (*p* = 0.015) and 100 mg (*p* = 0.02), versus celecoxib (Table S6).

### 3.2 Safety

Overall, 364 (32.0%) patients experienced 793 TEAEs. The TEAE rate was highest in the tramadol group, with 82 (39.4%) patients experiencing 212 TEAEs, and lowest in the CTC 150‐mg group, in which 60 (29.3%) patients experienced 132 TEAEs. Among all active treatment groups, the number of TEAEs was lowest in the CTC 200‐mg group, with 62 (29.8%) patients experiencing 108 AEs (Table 2).

**TABLE 2:** Summary of TEAEs (safety analysis set)

Event, *n* (%)	CTC 100 mg (*N =* 207)	CTC 150 mg (*N =* 205)	CTC 200 mg (*N =* 208)	Tramadol 100 mg (*N =* 208)	Celecoxib 100 mg (*N =* 206)	Placebo (*N =* 102)	Total (*N =* 1136)
TEAEs	63 (30.4)	60 (29.3)	62 (29.8)	82 (39.4)	67 (32.5)	30 (29.4)	364 (32.0)
Study drug‐related TEAEs	30 (14.5)	20 (9.8)	30 (14.4)	49 (23.6)	36 (17.5)	12 (11.8)	177 (15.6)
Severe TEAEs	1 (0.5)	2 (1.0)	1 (0.5)	4 (1.9)	3 (1.5)	1 (1.0)	12 (1.1)
Study drug‐related severe TEAEs	–	1 (0.5)	–	1 (0.5)	1 (0.5)	–	3 (0.3)
Serious TEAEs	2 (1.0)	3 (1.5)	–	3 (1.4)	3 (1.5)	–	11 (1.0)
Study drug‐related serious TEAEs	–	1 (0.5)	–	1 (0.5)	1 (0.5)	–	3 (0.3)
TEAEs leading to study discontinuation	2 (1.0)	1 (0.5)	4 (1.9)	6 (2.9)	4 (1.9)	–	17 (1.5)
TEAEs leading to death	–	–	–	–	–	–	–
Most frequent TEAEs (≥2%)							
Nausea	7 (3.4)	7 (3.4)	9 (4.3)	30 (14.4)	14 (6.8)	9 (8.8)	76 (6.7)
Somnolence	11 (5.3)	13 (6.3)	13 (6.3)	22 (10.6)	8 (3.9)	8 (7.8)	75 (6.6)
Constipation	10 (4.8)	12 (5.9)	10 (4.8)	22 (10.6)	6 (2.9)	5 (4.9)	65 (5.7)
Fatigue	11 (5.3)	10 (4.9)	8 (3.8)	6 (2.9)	12 (5.8)	9 (8.8)	56 (4.9)
Vomiting	2 (1.0)	7 (3.4)	7 (3.4)	16 (7.7)	11 (5.3)	4 (3.9)	47 (4.1)
Dizziness	7 (3.4)	7 (3.4)	7 (3.4)	12 (5.8)	5 (2.4)	5 (4.9)	43 (3.8)
Alanine aminotransferase increased	7 (3.4)	5 (2.4)	5 (2.4)	10 (4.8)	8 (3.9)	1 (1.0)	36 (3.2)
Gamma‐glutamyl transferase increased	4 (1.9)	6 (2.9)	4 (1.9)	9 (4.3)	8 (3.9)	1 (1.0)	32 (2.8)
Aspartate aminotransferase increased	5 (2.4)	4 (2.0)	3 (1.4)	6 (2.9)	6 (2.9)	1 (1.0)	25 (2.2)
Anaemia	2 (1.0)	4 (2.0)	6 (2.9)	5 (2.4)	3 (1.5)	1 (1.0)	21 (1.8)
Pruritus	2 (1.0)	3 (1.5)	4 (1.9)	6 (2.9)	3 (1.5)	2 (2.0)	20 (1.8)
Pyrexia	3 (1.4)	5 (2.4)	–	–	6 (2.9)	2 (2.0)	16 (1.4)

Abbreviations: CTC, co‐crystal of tramadol‐celecoxib; *n*, number of patients experiencing event. *N*, number of patients in analysis set; TEAE, treatment‐emergent adverse event.

Of the 793 TEAEs, 352 in 177 (15.6%) patients were considered to be study drug‐related. The rate was highest in the tramadol group, with 49 (23.6%) patients experiencing 114 treatment‐related TEAEs, and lowest in the CTC 150‐mg group, with 20 (9.8%) patients experiencing 38 treatment‐related TEAEs. Rates of severe TEAEs and of TEAEs leading to drug discontinuation were also highest in the tramadol group. The most common TEAE was nausea, followed by somnolence and constipation, all of which were most common in the tramadol group (Table 2, Figure S3). In total, 11 patients experienced serious TEAEs, most of which were not considered to be related to study drug, but instead related to the surgical intervention (Table 2). No clinically important changes in laboratory parameters, vital signs, or ECGs were observed, and no new or unexpected safety signals emerged for any treatment group. Patient‐reported OR‐SDS scores began at a low level and decreased throughout the study in all treatment groups, the only exception being constipation, which increased slightly from 4 to 120 h, particularly in the tramadol group. No treatment group showed particularly high scores.

### 3.3 Pharmacokinetics

Sparse exploratory PK analysis was performed in 196 patients. The observed concentrations of tramadol, *O*‐desmethyltramadol and celecoxib at each time point increased with higher CTC doses (Figure S4). The concentrations of all analytes were generally consistent with previous phase 1 data in healthy volunteers, with the exception of concentrations measured 2 h post‐dose (Videla et al., 2017, 2018).

## 4 DISCUSSION

The STARDOM2 trial evaluated the efficacy and safety of CTC for the treatment of acute moderate‐to‐severe pain following abdominal hysterectomy. In the prespecified analysis of the primary endpoint, SPID_0–4_, CTC 200 mg was superior to placebo and non‐inferior—but not superior—to tramadol. In one‐sided analyses of SPID_0–4_, CTC 150 and 200 mg were superior to placebo, while all CTC doses were non‐inferior to tramadol. Most patients did not use rescue medication (paracetamol) in the first 4 or 24 h after surgery. Mean daily paracetamol doses were highest in placebo and celecoxib groups and <500 mg in all groups.

There is an unmet need for new analgesic agents that provide effective pain relief while avoiding high levels of exposure to potent opioids. CTC contains racemic tramadol and celecoxib in a novel co‐crystal formulation that alters the physiochemical properties of both active constituents and optimizes their PK properties (Almansa et al., 2017; Cebrecos et al., 2021; Merlos et al., 2018; Port et al., 2019; Videla et al., 2018). This has been demonstrated in phase 1 studies. Following CTC administration, the maximum plasma concentration of tramadol is decreased, and the time to reach this maximum concentration is increased, compared with tramadol alone or when administered in free combination with celecoxib. The time to maximum celecoxib plasma concentration is shorter following administration of CTC than following administration of celecoxib alone. The decreased absorption of celecoxib that occurs when this drug is administered concomitantly with tramadol is minimized following CTC administration (Cebrecos et al., 2021; Videla et al., 2018). For example, in a phase 1 study, the maximum mean plasma concentration of celecoxib was 318 ng/ml after single‐dose administration of celecoxib 100 mg alone, 165 ng/ml when administered concomitantly with tramadol 100 mg, and 259 ng/ml after administration of CTC 200 mg (which includes 112 mg celecoxib) (Cebrecos et al., 2021).

These differences in PK might be responsible for an improved benefit/risk profile for CTC versus tramadol in trials conducted in patients with acute moderate‐to‐severe postoperative pain, including with respect to tolerability and the incidence of opioid‐related AEs. In a phase 2 trial following oral surgery, CTC showed superior efficacy to placebo and tramadol, with comparable tolerability to tramadol (López‐Cedrún et al., 2018). In the phase 3 STARDOM1 trial, conducted in Europe and Canada in patients who had undergone oral surgery, CTC had superior efficacy to tramadol and placebo, meeting the study's predefined primary objective (López‐Cedrún et al., 2022). In another phase 3 trial, SUSA‐301 (a full factorial study conducted in the USA), CTC demonstrated superior efficacy over tramadol, celecoxib and placebo following bunionectomy with osteotomy, meeting the study's primary objective (Viscusi et al., 2022). In both trials, the tolerability profile of CTC was consistent with that expected for tramadol and celecoxib, and no increase in the number or severity of side effects, or new safety signals, were observed for CTC versus active comparators.

The current study adds to this body of evidence regarding the benefit/risk profile for CTC and its emergence as a suitable treatment alternative for the management of acute moderate‐to‐severe pain. While CTC was not superior to tramadol in the prespecified gatekeeping analysis in STARDOM2, and thus the study did not achieve its primary endpoint, CTC 200 mg was non‐inferior to tramadol. Importantly, this was achieved with considerably lower total tramadol exposure versus treatment with tramadol 100 mg QID. Over the 120‐h observation period, CTC 200 mg (*rac*‐tramadol hydrochloride 88 mg/celecoxib 112 mg) BID resulted in a total cumulative tramadol dose of 880 mg. By contrast, tramadol 100 mg QID (the maximum licensed dose in Europe) gave rise to a cumulative tramadol dose of 2000 mg over the same period. The results of STARDOM2 thus suggest that at the recommended clinical dose of 200 mg BID, CTC can provide non‐inferior analgesia to tramadol alone, with considerably lower total opioid exposure.

Lower total tramadol exposure may also have contributed to the lower rates of TEAEs observed with CTC 200 mg BID compared with tramadol 100 mg QID. Treatment‐related TEAEs were reported by 14.4% of patients with CTC 200 mg versus 23.6% with tramadol. Nausea, somnolence and constipation were more common with tramadol than with CTC 200 mg, as were severe TEAEs and TEAEs leading to study drug discontinuation. Taken together, the results of STARDOM2 point to an improved benefit/risk profile for CTC 200 mg compared with tramadol alone: non‐inferior efficacy with considerably lower cumulative opioid exposure and lower rates of treatment‐related TEAEs. CTC has previously shown an improved benefit/risk profile over tramadol in phase 2 (López‐Cedrún et al. 2018) and phase 3 studies (López‐Cedrún et al., 2022; Viscusi et al., 2022). In all three studies, CTC 200 mg showed similar or improved tolerability to tramadol, in addition to superior efficacy.

Sparse PK sampling in this study showed lower plasma drug levels at 2 h post‐dose, compared with data from detailed PK profiles obtained in phase 1 studies (Videla et al., 2017, 2018). This may be due to the physiological status that occurs in open abdominal surgery, known as the ‘fluid storm’. Substantial fluid losses occur due to evaporation (Doherty & Buggy, 2012; Lamke et al., 1977). Bleeding and the richly irrigated nature of the uterus are also contributory factors (Santoso et al., 2001), and fluids (2 L crystalloids and 0.5–1 L colloids) are infused over a short period of time. Overall, fluid supply is favoured in the short term, which may explain the reduced drug levels observed at 2 h and the subsequent recovery to expected levels by 50 h post‐dose.

Well‐defined major abdominal surgery is an established model of acute moderate‐to‐severe visceral pain, and is recommended by the European Medicines Agency (EMA) and U.S. Food and Drug Administration (FDA) for the clinical evaluation of analgesics (European Agency for the Evaluation of Medicinal Products, 2016; U.S. Department of Health and Human Services, Food and Drug Administration, Center for Drug Evaluation and Research, 2014). In this regard, it is of value in the clinical assessment of analgesic agents, particularly as very few practical models of visceral pain are available. Intravenous tramadol also has a long history of use following this procedure (Alon et al., 1992; Coetzee & van Loggerenberg, 1998; Wilder‐Smith et al., 1999). In the current study, both active comparators were superior to placebo for the primary efficacy endpoint and the majority of additional endpoints, confirming that the study design was sensitive to differences in treatment effect. The study was adequately powered to detect superiority of CTC over tramadol, celecoxib and placebo and patient numbers were sufficient, as determined by sample size calculations. Nevertheless, there is some evidence to suggest that soft tissue surgeries such as abdominal hysterectomy may have lower assay sensitivity for demonstrating statistical significance compared with other pain models, such as bunionectomy or dental extraction (Singla et al., 2014). As reported by Singla et al., relative to other acute pain models, hysterectomy models are associated with a smaller effect size and lower recruitment rate, and require more participants to achieve 80% power (Singla, 2019). This may be reflected in the effect sizes seen in the current study, when considered in comparison with the placebo group. For example, 4‐h 30% responder rates were 44.2% (for CTC 200 mg), 38.0% (tramadol), 34.0% (celecoxib) and 30.4% (placebo). This may be reflective of differences in the types of anaesthesia and peri/postoperative medications used compared with other surgical pain models. Oral surgery, for example, is performed under local anaesthesia, whereas abdominal hysterectomy is performed under general anaesthesia and typically involves acute postoperative analgesia, most often intravenous opioids. These factors, along with differences in the typical age and comorbidity profile of patients undergoing each procedure, may make abdominal hysterectomy a less sensitive model than oral surgery for detecting differences in analgesic efficacy. This might explain why CTC showed non‐inferior efficacy to tramadol in STARDOM2 despite demonstrating superior efficacy in other surgical models. Future studies could investigate the reasons for the lower sensitivity/increased variability of abdominal hysterectomy versus other forms of surgery.

The use of general anaesthesia and acute peri/postoperative (intravenous opioid) analgesia may also have contributed to differences in AE rates observed between this study and studies in which CTC was administered after different surgical procedures (López‐Cedrún et al., 2022; Viscusi et al., 2022). Geographical variation in AE reporting—as has been noted in other conditions, such as rheumatoid arthritis—may also contribute (Keebler et al., 2020). Such variation may be due to cultural/societal factors, including relationships between patients and healthcare providers, access to healthcare and tolerance of pain and discomfort (Keebler et al., 2020).

STARDOM2 was a large, randomized, double‐blind, multicentre trial, including a placebo arm and two active comparator arms. The study examined the effects of study drugs on a range of measures, in an effort to capture the multidimensional nature of pain responses (Pogatzki‐Zahn et al., 2021), while applying a rigorous statistical approach to address the issue of multiple testing. Despite having observed a placebo effect, superiority to placebo was demonstrated. Primary and key secondary efficacy endpoints were formally evaluated using a predetermined parallel gatekeeping approach (Bretz et al., 2009). In this approach, the overall study alpha significance level was preserved, via performing multiple tests for several hypotheses in a sequential, hierarchical manner and splitting the significance level for testing the three CTC doses in parallel. Adjusted *p* values (for multiplicity) and CIs were derived for each hypothesis. This approach gave the trial a confirmatory nature, according to the requirements of the EMA and FDA (European Agency for the Evaluation of Medicinal Products, 2016; U.S. Department of Health and Human Services, Food and Drug Administration, Center for Drug Evaluation and Research, 2014). As ratings of pain intensity are subjective, with substantial inter‐ and intra‐individual variability, consistency across a range of outcomes, as was seen in STARDOM2, may be more clinically relevant than any single result.

We have noted the declining use of abdominal hysterectomy in clinical practice. However, abdominal hysterectomy is a model of nociceptive, inflammatory and visceral pain, and our findings are therefore applicable to surgery more generally. Indeed, by allowing the use of a range of surgical approaches to abdominal hysterectomy, and elective hysterectomy for a variety of benign indications, STARDOM2 mirrors real‐world clinical practice for this surgery, increasing the generalizability of the findings.

The ongoing opioid crisis in the United States highlights the need for new treatment options for acute pain that offer improved tolerability and reduced risk of dependence compared with high doses of potent opioids. The situation in Europe is also of concern, with increasing opioid prescriptions and opioids involved in most drug‐related deaths (European Monitoring Centre for Drugs and Drug Addiction, 2021). Although tramadol is not without its side effects, such as the risk of serotonin syndrome in some patient populations, it is associated with reduced levels of those side effects most commonly associated with potent opioids, such as respiratory depression (Edinoff et al., 2021). As a schedule IV opioid, tramadol is considered by the FDA and the World Health Organization to have lower potential for abuse or dependence, although the risk of abuse should still be considered. Multimodal analgesia is now recognized as best practice (As‐Sanie et al., 2017; Azari et al., 2013; Chou et al., 2016; Gan, 2017), and by including celecoxib in its co‐crystal structure, CTC targets an additional mechanism of analgesia beyond those of tramadol. Celecoxib has also been reported to have opioid‐sparing effects in postoperative pain management following hysterectomy (Ulm et al., 2018). NSAIDs have been known to cause gastrointestinal side effects in some patients and are typically associated with long‐term use. A large randomized trial showed that celecoxib (a selective cyclooxygenase‐2 inhibitor) has a lower rate of gastrointestinal effects than ibuprofen or naproxen (Nissen et al., 2016). Since CTC is intended for acute pain management, these celecoxib‐specific risks are not expected to be any greater than when celecoxib is used alone. No potentiation of tramadol or celecoxib AEs, or new safety signals, were observed in CTC clinical trials (López‐Cedrún et al., 2022; Viscusi et al., 2022). The current study also demonstrates that AEs that can be associated with tramadol use, such as constipation, may be lower with CTC than with tramadol alone.

In conclusion, in the prespecified analysis of the primary efficacy endpoint, SPID_0‐4_, CTC was not superior to tramadol in STARDOM2 but showed non‐inferior efficacy that was sustained throughout the 120‐h period, with a total cumulative tramadol dose of 880 mg compared with 2000 mg for tramadol alone. The rate of treatment‐related TEAEs was also lower with CTC than with tramadol. This safety profile, combined with non‐inferior efficacy despite considerably lower total opioid exposure, shows that CTC 200 mg has a clinically relevant improved benefit/risk profile compared with tramadol alone.

## AUTHOR CONTRIBUTIONS

RL, JF and SA contributed to study design. JF, SA, GR and LB‐H contributed to data collection. RL, AM, MS, JC, AV, EO, JF, SA, NG and CP‐S contributed to data analysis or interpretation. All authors discussed the results and commented on the manuscript, reviewed and critically revised the manuscript, approved the final draft and agree to be accountable for the accuracy and integrity of the work.

## FUNDING INFORMATION

The study was sponsored by Mundipharma Research GmBH & Co. KG (Limburg, Germany). ESTEVE Pharmaceuticals S.A. (Barcelona, Spain) invented and co‐developed CTC and performed the bioanalytical analysis. CTC is now under development by ESTEVE Pharmaceuticals S.A. Scientists employed by the funder and ESTEVE Pharmaceuticals S.A. participated in the design and conduct of the study, data review and interpretation and drafting of the article. All authors had full access to all study data and had final responsibility for the decision to submit for publication.

## DATA SHARING STATEMENT

ESTEVE Pharmaceuticals S.A. will consider requests for de‐identified patient‐level data and supporting study documents from qualified external researchers. Approval of requests will be at the discretion of ESTEVE Pharmaceuticals S.A. and will depend on the scientific merit of the proposed research and intended use of the data. If approval is granted, a Data Sharing Agreement must be signed and access to data will be provided only if ESTEVE Pharmaceuticals S.A. has legal authority to provide the data and there are no contradictory requirements relating to regulatory filings or reviews. Proposals should be sent to esteve@esteve.com.

## CONFLICT OF INTEREST

RL has received fees for consultancy and speaker activities from Pfizer, Eli Lilly, Compass, GSK, Avenue Therapeutics, MedinCell, Heron, Camurus, BioQ Pharma and Sintetica. AM, MS, JC, AV, EO and NG are employees of ESTEVE Pharmaceuticals. CP‐S was an employee of ESTEVE Pharmaceuticals and has pending or issued patents relevant to CTC. JF and SA were employees of Mundipharma Research Limited at the time of study. GR and LB‐H have no conflicts to declare.

## Supporting information


Appendix S1

## References

[ejp2021-bib-0001] Almansa, C. , Mercè, R. , Tesson, N. , Farran, J. , Tomàs, J. , & Plata‐Salamán, C. R. (2017). Co‐crystal of tramadol hydrochloride–celecoxib (CTC): A novel API–API co‐crystal for the treatment of pain. Crystal Growth & Design, 17, 1884–1892. 10.1021/acs.cgd.6b01848

[ejp2021-bib-0002] Alon E. , Atanassoff P. G. , & Biro P. (1992). Intravenous postoperative pain management using nalbuphine and tramadol. A combination of continuous infusion and patient‐controlled administration. Anaesthesist, 41, 83–87. (article in German) 1562097

[ejp2021-bib-0003] As‐Sanie, S. , Till, S. R. , Mowers, E. L. , Lim, C. S. , Skinner, B. D. , Fritsch, L. , Tsodikov, A. , Dalton, V. K. , Clauw, D. J. , & Brummett, C. M. (2017). Opioid prescribing patterns, patient use, and postoperative pain after hysterectomy for benign indications. Obstetrics & Gynecology, 130, 1261–1268. 10.1097/aog.0000000000002344 29112660 PMC5803559

[ejp2021-bib-0004] Azari, L. , Santoso, J. T. , & Osborne, S. E. (2013). Optimal pain management in total abdominal hysterectomy. Obstetrical & Gynecological Survey, 68, 215–227. 10.1097/OGX.0b013e31827f5119 23945838

[ejp2021-bib-0005] Bretz, F. , Maurer, W. , Brannath, W. , & Posch, M. (2009). A graphical approach to sequentially rejective multiple test procedures. Statistics in Medicine, 28, 586–604. 10.1002/sim.3495 19051220

[ejp2021-bib-0006] Cebrecos, J. , Carlson, J. D. , Encina, G. , Lahjou, M. , Sans, A. , Sust, M. , Vaque, A. , Morte, A. , Gascon, N. , & Plata‐Salamán, C. (2021). Celecoxib‐tramadol co‐crystal: A randomized 4‐way crossover comparative bioavailability study. Clinical Therapeutics, 43, 1051–1065. 10.1016/j.clinthera.2021.04.002 34167827

[ejp2021-bib-0007] Chou, R. , Gordon, D. B. , de Leon‐Casasola, O. A. , Rosenberg, J. M. , Bickler, S. , Brennan, T. , Carter, T. , Cassidy, C. L. , Chittenden, E. H. , Degenhardt, E. , Griffith, S. , Manworren, R. , McCarberg, B. , Montgomery, R. , Murphy, J. , Perkal, M. F. , Suresh, S. , Sluka, K. , Strassels, S. , Thirlby, R. , Viscusi, E. , Walco, G. A. , Warner, L Weisman, SJ , Wu, CL , Wu, C. L. (2016). Management of postoperative pain: A clinical practice guideline from the American pain society, the American Society of Regional Anesthesia and Pain Medicine, and the American Society of Anesthesiologists' Committee on Regional Anesthesia, Executive Committee, and Administrative Council. Journal of Pain, 17, 131–157. 10.1016/j.jpain.2015.12.008 26827847

[ejp2021-bib-0008] Coetzee, J. F. , & van Loggerenberg, H. (1998). Tramadol or morphine administered during operation: A study of immediate postoperative effects after abdominal hysterectomy. British Journal of Anaesthesia, 81, 737–741. 10.1093/bja/81.5.737 10193286

[ejp2021-bib-0009] Doherty, M. , & Buggy, D. J. (2012). Intraoperative fluids: How much is too much? British Journal of Anaesthesia, 109, 69–79. 10.1093/bja/aes171 22661747

[ejp2021-bib-0010] Edinoff, A. N. , Kaplan, L. A. , Khan, S. , Petersen, M. , Sauce, E. , Causey, C. D. , Cornett, E. M. , Imani, F. , Moradi, M. O. , Kaye, A. M. , & Kaye, A. D. (2021). Full opioid agonists and tramadol: Pharmacological and clinical considerations. Anesthesiology and Pain Medicine, 11, e119156. 10.5812/aapm.119156 34692448 PMC8520671

[ejp2021-bib-0011] European Agency for the Evaluation of Medicinal Products . (2016). Guideline on the clinical development of medicinal products intended for the treatment of pain. Retrieved from https://www.ema.europa.eu/en/documents/scientific‐guideline/guideline‐clinical‐development‐medicinal‐products‐intended‐treatment‐pain‐first‐version_en.pdf

[ejp2021-bib-0012] European Monitoring Centre for Drugs and Drug Addiction . (2021). Drug‐related deaths and mortality in Europe: Update from the EMCDDA expert network. Technical report. Publications Office of the European Union. Retrieved from https://www.emcdda.europa.eu/system/files/publications/13762/TD0221591ENN.pdf

[ejp2021-bib-0013] Gan, T. J. (2017). Poorly controlled postoperative pain: Prevalence, consequences, and prevention. Journal of Pain Research, 10, 2287–2298. 10.2147/JPR.S144066 29026331 PMC5626380

[ejp2021-bib-0014] Gan, T. J. , Habib, A. S. , Miller, T. E. , White, W. , & Apfelbaum, J. L. (2014). Incidence, patient satisfaction, and perceptions of post‐surgical pain: Results from a US national survey. Current Medical Research and Opinion, 30, 149–160. 10.1185/03007995.2013.860019 24237004

[ejp2021-bib-0015] Gascon, N. , Almansa, C. , Merlos, M. , Miguel, V. J. , Encina, G. , Morte, A. , Smith, K. , & Plata‐Salamán, C. (2019). Co‐crystal of tramadol‐celecoxib: Preclinical and clinical evaluation of a novel analgesic. Expert Opinion on Investigational Drugs, 28, 399–409. 10.1080/13543784.2019.1612557 31023091

[ejp2021-bib-0016] Keebler, D. , Teng, E. , Chia, J. , Galanter, J. , Peake, J. , & Tuckwell, K. (2020). Regional variations in adverse event reporting rates and ACR responses in placebo/standard‐of‐care arms of rheumatoid arthritis trials. Rheumatology, 59, 3023–3031. 10.1093/rheumatology/keaa043 32182362 PMC7516100

[ejp2021-bib-0017] Lamke, L. O. , Nilsson, G. E. , & Reithner, H. L. (1977). Water loss by evaporation from the abdominal cavity during surgery. Acta Chirurgica Scandinavica, 143, 279–284. 596094

[ejp2021-bib-0018] López‐Cedrún, J. , Videla, S. , Burgueno, M. , Juarez, I. , Aboul‐Hosn, S. , Martin‐Granizo, R. , Grau, J. , Puche, M. , Gil‐Diez, J. L. , Hueto, J. A. , Vaque, A. , Sust, M. , Plata‐Salamán, C. , Monner, A. , & Co‐Crystal of Tramadol‐Celecoxib Team . (2018). Co‐crystal of tramadol‐celecoxib in patients with moderate to severe acute post‐surgical oral pain: A dose‐finding, randomised, double‐blind, placebo‐ and active‐controlled, multicentre, phase II trial. Drugs in R & D, 18, 137–148. 10.1007/s40268-018-0235-y 29799099 PMC5995791

[ejp2021-bib-0019] López‐Cedrún J. L. , Kowalik S. , Bescós S. , Fettiplace J. , Morte A. , González D. , Cebrecos J. , Gascón N. , & Plata‐Salamán C. (2022, April). Efficacy and safety of co‐crystal of tramadol‐celecoxib versus tramadol and placebo in moderate‐to‐severe acute post‐surgical oral pain: A multicentre, randomised, double‐blind, phase 3 trial (STARDOM1). Presented at The 12th Congress of the European Pain Federation, Dublin, Ireland.

[ejp2021-bib-0020] Merlos, M. , Portillo‐Salido, E. , Brenchat, A. , Aubel, B. , Buxens, J. , Fisas, A. , Codony, X. , Romero, L. , Zamanillo, D. , & Vela, J. M. (2018). Administration of a co‐crystal of tramadol and celecoxib in a 1:1 molecular ratio produces synergistic antinociceptive effects in a postoperative pain model in rats. European Journal of Pharmacology, 833, 370–378. 10.1016/j.ejphar.2018.06.022 29932927

[ejp2021-bib-0021] Nissen, S. E. , Yeomans, N. D. , Solomon, D. H. , Luscher, T. F. , Libby, P. , Husni, M. E. , Graham, D. Y. , Borer, J. S. , Wisniewski, L. M. , Wolski, K. E. , Wang, Q. , Menon, V. , Ruschitzka, F. , Gaffney, M. , Beckerman, B. , Berger, M. F. , Bao, W. , Lincoff, A. M. , & P. T. Investigators . (2016). Cardiovascular safety of celecoxib, naproxen, or ibuprofen for arthritis. The New England Journal of Medicine, 375, 2519–2529. 10.1056/NEJMoa1611593 27959716

[ejp2021-bib-0022] Pogatzki‐Zahn, E. M. , Liedgens, H. , Hummelshoj, L. , Meissner, W. , Weinmann, C. , Treede, R. D. , Vincent, K. , Zahn, P. , Kaiser, U. , & IMI‐PainCare PROMPT Consensus Panel . (2021). Developing consensus on core outcome domains for assessing effectiveness in perioperative pain management: Results of the PROMPT/IMI‐PainCare Delphi meeting. Pain, 162, 2717–2736. 10.1097/j.pain.0000000000002254 34181367

[ejp2021-bib-0023] Port, A. , Almansa, C. , Enrech, R. , Bordas, M. , & Plata‐Salamán, C. R. (2019). Differential solution behavior of the new API–API co‐crystal of tramadol–celecoxib (CTC) versus its constituents and their combination. Crystal Growth & Design, 19, 3172–3182. 10.1021/acs.cgd.9b00008

[ejp2021-bib-0024] Santoso, J. T. , Dinh, T. A. , Omar, S. , Gei, A. F. , & Hannigan, E. V. (2001). Surgical blood loss in abdominal hysterectomy. Gynecologic Oncology, 82, 364–366. 10.1006/gyno.2001.6269 11531295

[ejp2021-bib-0025] Sinatra, R. (2010). Causes and consequences of inadequate management of acute pain. Pain Medicine, 11, 1859–1871. 10.1111/j.1526-4637.2010.00983.x 21040438

[ejp2021-bib-0026] Singla N. K. (2019, November). Acute vs chronic pain: Experimental models, regulatory pathways and market opportunities. Paper presented at American Pain Society Conference on Analgesic Trials, Milwaukee, Wisconsin.

[ejp2021-bib-0027] Singla, N. K. , Desjardins, P. J. , & Chang, P. D. (2014). A comparison of the clinical and experimental characteristics of four acute surgical pain models: Dental extraction, bunionectomy, joint replacement, and soft tissue surgery. Pain, 155, 441–456. 10.1016/j.pain.2013.09.002 24012952

[ejp2021-bib-0028] U.S. Department of Health and Human Services, Food and Drug Administration, Center for Drug Evaluation and Research (2014). Guidance for industry. Analgesic Indications: Developing Drug and Biological Products. Retrieved from https://www.fdanews.com/ext/resources/files/02/02‐05‐14‐Analgesic.pdf

[ejp2021-bib-0029] Ulm, M. A. , ElNaggar, A. C. , & Tillmanns, T. D. (2018). Celecoxib versus ketorolac following robotic hysterectomy for the management of postoperative pain: An open‐label randomized control trial. Gynecologic Oncology, 151, 124–128. 10.1016/j.ygyno.2018.08.015 30121131

[ejp2021-bib-0030] Videla, S. , Lahjou, M. , Vaque, A. , Sust, M. , Encabo, M. , Soler, L. , Sans, A. , Sicard, E. , Gascon, N. , Encina, G. , & Plata‐Salamán, C. (2017). Single‐dose pharmacokinetics of co‐crystal of tramadol‐celecoxib: Results of a four‐way randomized open‐label phase I clinical trial in healthy subjects. British Journal of Clinical Pharmacology, 83, 2718–2728. 10.1111/bcp.13395 28810061 PMC5698592

[ejp2021-bib-0031] Videla, S. , Lahjou, M. , Vaque, A. , Sust, M. , Escriche, M. , Soler, L. , Sans, A. , Sicard, E. , Gascon, N. , Encina, G. , & Plata‐Salamán, C. (2018). Pharmacokinetics of multiple doses of co‐crystal of tramadol‐celecoxib: Findings from a four‐way randomized open‐label phase I clinical trial. British Journal of Clinical Pharmacology, 84, 64–78. 10.1111/bcp.13428 28888220 PMC5736845

[ejp2021-bib-0032] Viscusi E. R. , de Leon‐Casasola O. , Cebrecos J. , Jacobs A. , Morte A. , Ortiz E. , Sust M. , Vaqué A. , Gottlieb I. , Daniels S. , Gimbel J. S. , Muse D. , Winkle P. , Kuss M. E. , Videla S. , Gascón N. , & Plata‐Salamán C. (2022). Celecoxib‐tramadol co‐crystal in patients with moderate‐to‐severe pain following bunionectomy with osteotomy: A phase 3, randomized, double‐blind, factorial, active‐ and placebo‐controlled trial. Pain Practice. 10.1111/papr.13136 PMC10084286 35686380

[ejp2021-bib-0033] Wilder‐Smith, C. H. , Hill, L. , Wilkins, J. , & Denny, L. (1999). Effects of morphine and tramadol on somatic and visceral sensory function and gastrointestinal motility after abdominal surgery. Anesthesiology, 91, 639–647. 10.1097/00000542-199909000-00013 10485772

