# Supplementary material for: Efficacy and safety of co‐crystal of tramadol‐celecoxib (CTC) in acute moderate‐to‐severe pain after abdominal hysterectomy: A randomized, double‐blind, phase 3 trial (STARDOM2)
Source: Eur J Pain. 2022 Sep 24;26(10):2083–96. doi: 10.1002/ejp.2021 (PMC9826359; doi:10.1002/ejp.2021)

SUPPLEMENTARY MATERIALS

Efficacy and safety of co-crystal of tramadol-celecoxib (CTC) in acute moderate-to-severe pain after abdominal hysterectomy: A randomized, double-blind, phase 3 trial (STARDOM2)

Richard Langford, Adelaida Morte, Mariano Sust, Jesús Cebrecos, Anna Vaqué, Esther Ortiz, James Fettiplace, Shola Adeyemi, Grzegorz Raba, Liudmila But-Husaim, Neus Gascón, Carlos Plata-Salamán

CONTENTS

SUPPLEMENTARY METHODS 3
Inclusion and exclusion criteria 3
Inclusion criteria 3
Exclusion criteria 4
Permitted and prohibited preoperative, perioperative, and postoperative medications 6
Permitted 6
Prohibited 7
Additional secondary endpoints 8
Sum of pain intensity differences over 0–12, 0–24, 0–48, 0–72, 0–96, and 0–120 hours 8
Total pain relief over 0–4, 0–12, 0–24, 0–48, 0–72, 0–96, and 0–120 hours 9

Treatment response (30% and 50%) ... 9

Time to perceptible pain relief and time to meaningful pain relief ... 9

Rescue medication use ... 10

Supplementary Tables and Figures ... 11

Table S1 Countries and study centres of patients who completed the study ... 11

Table S2 Evaluation of primary and key secondary hypotheses using the parallel gatekeeping procedure ... 13

Table S3 Sum of pain intensity differences over 0–12, 0–24, 0–48, 0–72, 0–96, and 0–120 hours (last observation carried forward, full analysis set) ... 16

Table S4 Total pain relief over 0–4, 0–12, 0–24, 0–48, 0–72, 0–96, and 0–120 hours (last observation carried forward, full analysis set) ... 18

Table S5 Responder rates, perceptible pain relief and meaningful pain relief (full analysis set) ... 20

Table S6 Use of rescue medication (full analysis set) ... 22

Fig. S1 Study design ... 23

Fig. S2 Patient disposition. ... 24

Fig. S3 Summary of (a) TEAEs and study-drug-related TEAEs and (b) the most frequently occurring TEAEs (safety analysis set). ... 25

Fig. S4. Plasma drug levels (PK analysis set): (a) tramadol plasma concentration data (mean ± SD), (b) O-desmethyltramadol plasma concentration data (mean ± SD) and (c) celecoxib plasma concentration data (mean ± SD) ... 26

SUPPLEMENTARY METHODS

Inclusion and exclusion criteria

Inclusion criteria

Patients who met all of the following criteria were included in the study:

1. Female patients ≥18 years old on the day of consent.
2. Willing and able to provide informed consent.
3. Scheduled to have a total or subtotal abdominal hysterectomy under general anaesthesia via a Pfannenstiel incision.
4. The elective procedure (total or subtotal hysterectomy with or without salpingo-oophorectomy) had to be for benign conditions within 28 days of screening. Patients with stage 0 carcinoma in situ of cervix, endometrial hyperplasia, or clinically staged 1A or 1B endometrial cancer were allowed to participate.
5. American Society of Anesthesiology physical status of I or II.
6. Women of childbearing potential had to use highly effective methods of contraception throughout the study.
7. Good general health as judged by investigators on the basis of medical history and physical examination.
8. Willingness to comply with the study procedures and requirements.

Additional inclusion criteria after surgery:

1. Abdominal hysterectomy completed without any immediate complication.
2. Tolerating oral fluids, no uncontrolled nausea/vomiting, and ready to take oral analgesia.
3. Patient was alert and calm, and spontaneously paid attention to the caregiver, e.g. Richmond Agitation-Sedation Scale = 0.
4. Patient with moderate or severe pain (qualifying pain intensity–visual analogue scale [PI-VAS] score ≥45 mm and <70 mm or ≥70 mm) as a result of a surgical procedure (abdominal hysterectomy) under general anaesthesia. This had to be measured within a maximum of 30 hours after end of surgery

and patients could only be randomized on the day after surgery, after cessation of postoperative analgesia.

Exclusion criteria

Patients who met any of the following criteria were excluded from the study:

1. Any abnormal laboratory value that was clinically significant (in the opinion of the investigator) that would compromise the safety of the patient in the study.
2. Any recent history of frequent nausea or vomiting, or dizziness within the last 3 months regardless of aetiology.
3. Any medical condition or treatment that was either a warning or contraindication as per the summary of product characteristics for tramadol (e.g. selective serotonin reuptake inhibitors [SSRIs], serotonin-norepinephrine reuptake inhibitors [SNRIs], tricyclic antidepressants, monoamine oxidase [MAO] inhibitors [within 14 days before taking a study drug], antipsychotics, anticonvulsants, and other seizure threshold-lowering medicinal products), celecoxib (e.g. increased risk of postoperative bleeding, active peptic ulceration, gastrointestinal bleeding, or inflammatory bowel disease), or paracetamol.
4. Known sensitivity and/or contraindication to tramadol, celecoxib, paracetamol, sulphonamides, opioids, non-steroidal anti-inflammatory drugs (NSAIDs), cyclooxygenase-2 inhibitors, or related compounds or formulation excipients, as well as severe hypersensitivity reactions (e.g. anaphylactic shock, bronchospasm, angio-oedema) to any drugs.
5. Known to have had inadequate pain relief from paracetamol, tramadol, or celecoxib.
6. Requiring any medication that was prohibited.
7. Patients who, in the investigator’s opinion, were considered at increased risk of operative (those associated with the surgical procedure and general anaesthesia) and postoperative complications, for example, excessive postoperative bleeding, infection.

8. Any history of drug or alcohol abuse, misuse, physical or psychological dependence, mood changes, sleep disturbance, and functional capacity that had an impact on pain perception.
9. Significant neurological or psychiatric disorders, including mental instability (unrelated to the pain) that could interfere with pain assessment; other pain that might impair the assessment of nociceptive pain.
10. Any history of significant and/or inadequately controlled cardiovascular (uncontrolled high blood pressure, high risk of cardiovascular events, severe heart failure), pulmonary, haematological (including coagulopathy/bleeding disorders), neurological (e.g. patients with epilepsy or those susceptible to seizures), hepatic disease (e.g. severe hepatic impairment), renal disease (e.g. serum creatinine level greater than 1.5 times the upper limit of normal, impaired renal function in patients taking diuretics, angiotensin-converting enzyme inhibitors, or angiotensin II antagonists), endocrine, immunological, dermatological painful conditions, or any other conditions that may compromise the ability of the patient to participate in the study or might interfere with drug absorption, distribution, metabolism, or excretion.
11. Previous randomization in this study.
12. Participation in a clinical research study involving a new chemical entity or an experimental drug within 30 days of study entry (defined as the start of the screening period).
13. Treated regularly with opioid analgesic or NSAIDs within 30 days prior to screening or who had received a long-acting NSAID within 3 days prior to the start of the surgery.
14. Incapable of complying with the protocol.
15. Epidural or spinal anaesthesia or infiltration of the wound with an infusion of a local anaesthetic agent was not allowed. A single perioperative dose was allowed.
16. History or ongoing chronic pelvic inflammatory disease or painful endometriosis.
17. History of advanced gynaecological cancers.

Additional exclusion criteria after surgery:

1. Serious complication during surgery and up to randomization, including:
    a. Postoperative primary and secondary bleed that could not be controlled.
    b. Patients in whom the abdominal hysterectomy surgery was not completed as planned.
2. Any factors that may, in the investigator's opinion, have affected compliance with the protocol.
3. Clinical need for antiemetics (apart from standard perioperative practice as defined in the protocol) or any other medication that is prohibited.
4. Received any analgesic medication other than perioperative analgesia as described in the protocol.
5. Any concerns that renal function had deteriorated, for example, a laboratory parameter, profound hypotension, poor urine output, or excessive bleeding during surgery.

Permitted and prohibited preoperative, perioperative, and postoperative medications

Permitted

Perioperative

- Modest dose of benzodiazepine premedication (e.g. intravenous [IV] midazolam 1–2 mg and temazepam 5–10 mg orally)
- IV induction with propofol
- Isoflurane, sevoflurane, or desflurane without nitrous oxide
- A single perioperative dose of local anaesthetic for wound infiltration was allowed. Lidocaine (lignocaine), articaine, and mepivacaine were allowed
- Bolus doses of IV fentanyl or morphine were permitted, as needed
- IV induction with paracetamol up to 1 g

- Metoclopramide, ondansetron, and prochlorperazine (e.g. stemetil) as needed were permitted for the treatment of nausea and vomiting
- Prophylactic doses of low molecular weight heparin
- Cephalosporins, amoxicillin, amoxicillin plus clavulanic acid, and clindamycin were permitted as prophylactic antibiotics, but all others should be confirmed with the medical monitor

Postoperative

- Treatment with IV morphine or fentanyl (by bolus, infusion, or patient-controlled pump) were allowed but needed to be stopped before start of PI-VAS measurements at Visit 3 to qualify for randomization
- Metoclopramide, ondansetron, and prochlorperazine (e.g. stemetil) as needed were permitted for the treatment of nausea and vomiting
- Prophylactic doses of low molecular weight heparin

Prohibited

Preoperative

- Regular opioid or NSAID analgesic medication
- Long-acting NSAIDs (piroxicam, sulindac, nabumetone, naproxen, meloxicam, celecoxib) within 3 days prior to the start of the surgery (exclusion criterion)

Perioperative

- Nitrous oxide
- Bupivacaine
- Epidural or spinal anaesthesia
- Opioid analgesic medication: In general, not allowed (including IV sufentanil and remifentanil), except IV morphine and fentanyl as detailed above
- Any NSAID
- Metamizole

- Dexamethasone/corticosteroids

Postoperative

- Epidural or spinal anaesthesia
- Non-opioid analgesic medication (except intravenous paracetamol)
- Opioid analgesic medication: In general, not allowed (including IV sufentanil and remifentanil) except intravenous morphine and fentanyl as detailed above
- Any NSAID
- Metamizole
- Dexamethasone/corticosteroids

During intake of study drug:

- Serotonergic drugs, including SSRIs and SNRIs
- Tricyclic antidepressants
- MAO inhibitors
- Antipsychotics
- Anticonvulsant and other seizure threshold-lowering medicinal products (e.g. bupropion, mirtazapine, tetrahydrocannabinol, N-methyl-D-aspartate antagonists)
- Opioids
- NSAIDs, including paracetamol (except for study pain rescue medication) and metamizole
- Acetylsalicylic acid (aspirin)
- Dexamethasone/corticosteroids

Additional secondary endpoints

Sum of pain intensity differences over 0–12, 0–24, 0–48, 0–72, 0–96, and 0–120 hours

The sum of pain intensity differences (SPID) was calculated at 12, 24, 48, 72, 96, and 120 hours based on PI-VAS values with last observation carried forward (LOCF) imputation applied. Statistical analyses of the secondary SPID endpoints were

performed using the same analysis of covariance (ANCOVA) model as in the primary analysis of the primary endpoint $SPID_{0–4}$ for the full analysis set and per-protocol analysis set.

Total pain relief over 0–4, 0–12, 0–24, 0–48, 0–72, 0–96, and 0–120 hours

Pain relief was calculated based on pain relief assessments completed by patients on a 5-point categorical scale. Total pain relief (TOTPAR) over 0–4, 0–12, 0–24, 0–48, 0–72, 0–96, and 0–120 hours was calculated based on the LOCF approach and were analysed using the same ANCOVA model applied for the primary endpoint $SPID_{0–4}$.

Treatment response (30% and 50%)

Patients with a reduction of pain, as assessed on the PI-VAS scale, of 50% from 0–4 hours were defined as 50% responders at 4 hours.

A treatment response of 50% at 12, 24, 48, 72, 96, and 120 hours, and a 30% treatment response at 4, 12, 24, 48, 72, 96, and 120 hours, were analysed using logistic regression models with treatment and qualifying pain intensity (QPI; moderate, severe) as fixed effects, centre (with pooling of centres applied) as a random effect, and pre-dose (0 hours) as a covariate.

For treatment response parameters, odds ratios and corresponding two-sided 95% confidence intervals for treatment differences were calculated, along with *p* values for two-sided tests of the null hypothesis.

Time to perceptible pain relief and time to meaningful pain relief

Patients used their electronic diaries to record time to perceptible pain relief (PPR) and time to meaningful pain relief (MPR). Time was measured from the time of the first intake of study medication. The patient stopped the timer for the first time as soon as they noted that their pain was not getting worse and had possibly improved. This was the time to PPR.

Once the patient began to feel clearly less pain, they stopped the timer for a second time. This was the time to MPR.

Time to PPR and time to MPR were summarized using Kaplan–Meier estimates and were analysed via time-to-event methodology using Cox proportional hazards models.

Rescue medication use

The following variables were derived from rescue medication (RM) use data: RM use in the first 4 hours, average number of RM use occasions per 24 hours, average dose of RM per 24 hours, and time to first RM use.

Average RM use and average dose (mg) of RM were summarized using descriptive statistics. Average dose of RM per 24 hours was analysed further using an ANCOVA model with treatment and QPI as fixed effects, centre (with pooling of centres applied) as a random effect, and pre-dose (0 hours) as a covariate.

Supplementary Tables and Figures

Table S1 Countries and study centres of patients who completed the study

**Country** *Study centre*	**Patients who completed study n (% of total who completed study)**	**Country** *Study centre*	**Patients who completed study n (% of total who completed study)**	**Country** *Study centre*	**Patients who completed study n (% of total who completed study)**
**Total**	1,066 (100)	*1706*	2 (<1)	*2712*	43 (4)
**Belarus**	149 (14)	*1707*	39 (4)	*2713*	28 (3)
401	38 (4)	*1708*	15 (1)	**Russia**	109 (10)
402	16 (2)	*1710*	4 (<1)	*3001*	1 (<1)
403	9 (1)	*1711*	3 (<1)	*3002*	11 (1)
404	75 (7)	*1712*	6 (1)	*3003*	10 (1)
405	11 (1)	*1714*	22 (2)	*3004*	5 (<1)
**Bulgaria**	254 (24)	*1715*	38 (4)	*3005*	8 (1)
601	88 (8)	*1718*	10 (1)	*3006*	1 (<1)
602	3 (<1)	**Latvia**	123 (12)	*3007*	17 (2)
603	13 (1)	*2101*	29 (3)	*3010*	7 (1)
604	6 (1)	*2102*	52 (5)	*3011*	8 (1)
605	26 (2)	*2103*	42 (4)	*3012*	2 (<1)
607	42 (4)	**Poland**	239 (22)	*3015*	39 (4)
608	7 (1)	*2701*	82 (8)	**Spain**	20 (2)
609	11 (1)	*2702*	2 (<1)	*3502*	1 (<1)
611	46 (4)	*2703*	0 (0)	*3503*	3 (<1)
612	11 (1)	*2704*	26 (2)	*3505*	2 (<1)
613	1 (<1)	*2705*	17 (2)	*3506*	5 (<1)
**Hungary**	172 (16)	*2706*	3 (<1)	*3508*	5 (<1)
1702	8 (1)	*2708*	17 (2)	*3509*	0 (0)
1703	11 (1)	*2709*	10 (1)	*3515*	2 (<1)
1704	14 (1)	*2710*	11 (1)	*3517*	2 (<1)

Percentages shown to nearest whole number.

Table S2 Evaluation of primary and key secondary hypotheses using the parallel gatekeeping procedure

Step	Hypothesis (analysis set)	Type of estimate	n_1, n_2 / N	Estimate	95% CI	Raw p value	Available α	Adjusted CI	Null hypothesis outcome (p < available α)
**Treatment: CTC 200 mg**									
1	H11: $SPID_{0–4}$ superiority of CTC vs placebo (FAS)[a]	Diff. LS means[b]	208, 102 / 1,138	–20.0	–34.4, –5.6	0.0032	0.0083	–37.6, –2.4	Rejected
2	H12: $SPID_{0–4}$ non-inferiority of CTC vs tramadol (PPAS)[a]	Diff. LS means[c]	150, 154 / 850	–1.7	–14.8, 11.3	0.0000	0.0083	–17.6, 14.2	Rejected
3	H13: $SPID_{0–4}$ superiority of CTC over tramadol (FAS)[a]	Diff. LS means[d]	208, 208 / 1,138	1.7	–9.9, 13.4	0.3849	0.0083	–12.5, –16.0	Accepted
4	H14: $SPID_{0–4}$ superiority of CTC over celecoxib (FAS)[a]	Diff. LS means[e]	208, 205 / 1,138	–1.5	–13.2, 10.2	0.3996	0.0000	–	–
5	H15: 50% responder at 4 h (FAS)[f]	OR[g]	208, 208 / 1,138	1.2	0.8, 1.9	0.2045	0.0000	–	–
6	H16: Use of rescue medication during first 4 h[f]	OR[h]	206, 207 / 1,138	0.9	0.5, 1.5	0.3001	0.0000	–	–
**Treatment: CTC 150 mg**									
7	H11: $SPID_{0–4}$ superiority of CTC vs placebo (FAS)[a]	Diff. LS means[b]	207, 102 / 1,138	–16.8	–31.2, –2.4	0.0109	0.0083	–34.4, 0.7	Accepted
8	H12: $SPID_{0–4}$ non-inferiority of CTC vs tramadol (PPAS)[a]	Diff. LS means[c]	157, 154 / 850	–0.2	–13.2, 12.7	0.0000	0.0000	–	–
9	H13: $SPID_{0–4}$ superiority of CTC over tramadol (FAS)[a]	Diff. LS means[d]	207, 208 / 1,138	4.9	–6.8, 16.6	0.2057	0.0000	–	–
10	H14: superiority of CTC over celecoxib (FAS)[a]	Diff. LS means[e]	207, 205 / 1,138	1.6	–10.1, 13.4	0.3922	0.0000	–	–
11	H15: 50% responder at 4 h (FAS)[f]	OR[g]	207, 208 / 1,138	1.0	0.6, 1.6	0.4933	0.0000	–	–
12	H16: Use of rescue medication during first 4 h[f]	OR[h]	206, 207 / 1,138	0.8	0.4, 1.3	0.1532	0.0000	–	–

Step	Hypothesis (analysis set)	Type of estimate	n_1, n_2 / N	Estimate	95% CI	Raw *p* value	Available α	Adjusted CI	Null hypothesis outcome ($p <$ available α)
**Treatment: CTC 100 mg**									
13	H11: $SPID_{0–4}$ superiority of CTC vs placebo (FAS)[a]	Diff. LS means[b]	207, 102 / 1,138	−10.8	−25.2, 3.5	0.0692	0.0083	−28.4, 6.7	Accepted
14	H12: $SPID_{0–4}$ non-inferiority of CTC vs tramadol (PPAS)[a]	Diff. LS means[c]	159, 154 / 850	7.8	−5.1, 20.6	0.0000	0.0000	–	–
15	H13: $SPID_{0–4}$ superiority of CTC over tramadol (FAS)[a]	Diff. LS means[d]	207, 208 / 1,138	10.9	−0.8, 22.6	0.0341	0.0000	–	–
16	H14: superiority of CTC over celecoxib (FAS)[a]	Diff. LS means[e]	207, 205 / 1,138	7.6	−4.1, 19.3	0.1003	0.0000	–	–
17	H15: 50% responder at 4 h (FAS)[f]	OR[g]	207, 208 / 1,138	0.7	0.4, 1.2	0.0999	0.0000	–	–
18	H16: Use of rescue medication during first 4 h[f]	OR[h]	206, 207 / 1,138	1.1	0.7, 1.9	0.2971	0.0000	–	–

Adjusted CIs were based on the available α for a given hypothesis. Evaluation of the null hypotheses presented in this table are based on the available α from the relevant step of the gatekeeping procedure. For CTC 200 mg, the first two hypotheses (superiority over placebo in the FAS and non-inferiority vs tramadol in the PPAS) were rejected, and the third hypothesis (superiority over tramadol in the FAS) was accepted. For CTC 150 mg and 100 mg, the first hypothesis (superiority over placebo in the FAS) was accepted.

[a]$SPID_{0–4}$ analysed with an ANCOVA model with treatment and QPI at randomization (moderate or severe) as fixed effects, pooled centre as a random effect, and pre-dose (0 h) pain intensity as a covariate.

[b]$SPID_{0–4}$ diff. LS means of placebo–CTC; *p* value from a one-sided test (upper limit) of the null hypothesis that the difference is ≥0 mm·h.

[c]$SPID_{0–4}$ diff. LS means of tramadol–CTC; *p* value from one-sided test (upper limit) of the null hypothesis that the difference is ≥40 mm·h.

[d]$SPID_{0–4}$ diff. LS means of tramadol–CTC; *p* value from a one-sided test (upper limit) of the null hypothesis that the difference is ≥0 mm·h.

[e]$SPID_{0–4}$ diff. LS means of celecoxib–CTC; *p* value from a one-sided test (upper limit) of the null hypothesis that the difference is ≥0 mm·h.

[f]Data were analysed using a logistic regression model with treatment and QPI at randomization (moderate or severe) as fixed effects, pooled centre as a random effect, and pre-dose (0 h) pain intensity as a covariate.

[g]OR for observing a 50% response at 4 h for CTC versus tramadol; *p* value from one-sided test (lower limit) of the null hypothesis that the OR is ≤1.

[h]OR for using ≥1 dose of rescue medication during the first 4 h for CTC versus tramadol; *p* value from one-sided test (upper limit) of the null hypothesis that the OR is ≤1.

ANCOVA, analysis of covariance; CI, confidence interval; CTC, co-crystal of tramadol-celecoxib; diff. LS means, difference in least-squares means; FAS, full analysis set; *N*, number of patients in analysis set; n_1, number of patients used for analysis in CTC treatment arm; n_2, number of patients used for analysis in placebo or tramadol arm; OR, odds ratio; PPAS, per-protocol analysis set; QPI, qualifying pain intensity; $SPID_{0–4}$, sum of pain intensity differences over 0–4 h.

Table S3 Sum of pain intensity differences over 0–12, 0–24, 0–48, 0–72, 0–96, and 0–120 hours (last observation carried forward, full analysis set)

		CTC 100 mg (*n* = 207)	CTC 150 mg (*n* = 207)	CTC 200 mg (*n* = 208)	Tramadol 100 mg (*n* = 208)	Celecoxib 100 mg (*n* = 206)	Placebo (*n* = 102)
**0–12 h**	Mean (SD)	205.2 (232.3)	209.6 (230.6)	238.4 (233.3)	262.1 (242.1)	207.3 (245.3)	154.0 (205.6)
	Diff. vs placebo[a]	−41.6 (−90.0, 6.9) [0.093]	−68.3 (−116.9, −19.7) [0.006]	−82.5 (−131.1, −34.0) [<0.001]			
	Diff. vs tramadol[a]	51.2 (11.7, 90.8) [0.011]	24.5 (−15.1, 64.0) [0.225]	10.3 (−29.2, 49.7) [0.610]			
	Diff. vs celecoxib[a]	10.2 (−29.3, 49.7) [0.613]	−16.6 (−56.4, 23.2) [0.414]	−30.8 (−70.3, 8.8) [0.127]			
**0–24 h**	Mean (SD)	476.1 (517.3)	469.9 (505.0)	508.5 (514.2)	588.9 (518.5)	440.6 (543.9)	330.1 (461.6)
	Diff. vs placebo[a]	−130.2 (−237.7, −22.7) [0.018]	−170.9 (−278.7, −63.1) [0.002]	−180.9 (−288.6, −73.2) [0.001]			
	Diff. vs tramadol[a]	101.9 (14.2, 189.6) [0.023]	61.3 (−26.5, 149.0) [0.171]	51.2 (−36.3, 138.8) [0.251]			
	Diff. vs celecoxib[a]	−18.1 (−105.7, 49.7) [0.686]	−58.7 (−147.0, 29.6) [0.192]	−68.8 (−156.5, 18.9) [0.124]			
**0–48 h**	Mean (SD)	1,091.4 (1,144.9)	1,083.5 (1,095.0)	1,166.2 (1,123.0)	1,337.2 (1,101.8)	980.7 (1,178.3)	791.7 (1,062.0)
	Diff. vs placebo[a]	−274.7 (−510.6, −38.8) [0.023]	−359.3 (−595.8, −122.7) [0.003]	−389.1 (−625.4, −152.9) [0.001]			
	Diff. vs tramadol[a]	223.8 (31.3, 416.3) [0.023]	139.3 (−53.3, 331.8) [0.156]	109.4 (−82.8, 301.5) [0.264]			
	Diff. vs celecoxib[a]	−75.4 (−267.6, 116.9) [0.442]	−159.9 (−353.7, 33.8) [0.106]	−189.8 (−382.3, 2.7) [0.053]			
**0–72 h**	Mean (SD)	1,787.5 (1,809.3)	1,777.3 (1,721.5)	1,915.6 (1,777.9)	2,167.0 (1,717.3)	1,609.9 (1,853.6)	1,371.7 (1,713.2)
	Diff. vs placebo[a]	389.3 (−762.6, −16.1) [0.041]	−515.5 (−889.8, −141.2) [0.007]	−581.4 (−955.3, −207.4) [0.002]			
	Diff. vs tramadol[a]	345.3 (40.7, 649.9) [0.026]	219.1 (−85.5, 523.8) [0.158]	153.3 (−150.8, 457.3) [0.323]			
	Diff. vs celecoxib[a]	−125.8 (−430.0, 178.4) [0.417]	−252.0 (−558.5, 54.6) [0.107]	−317.8 (−622.4, −13.3) [0.041]			
**0–96 h**	Mean (SD)	2,535.5 (2,500.5)	2,544.1 (2,386.4)	2,723.4 (2,465.8)	3,053.6 (2,349.1)	2,313.4 (2,571.8)	2,046.7 (2,415.0)

	Diff. vs placebo[a]	−471.1 (−989.3, 47.0) [0.075]	−657.9 (−1,177.5, −138.3) [0.013]	−744.9 (−1,263.9, −225.8) [0.005]			
	Diff. vs tramadol[a]	473.4 (50.5, 896.2) [0.028]	286.6 (−136.3, 709.5) [0.184]	199.7 (−222.4, 621.7) [0.353]			
	Diff. vs celecoxib[a]	−155.9 (−578.2, 266.4) [0.469]	−342.7 (−768.3, 82.9) [0.114]	−429.6 (−852.5, −6.8) [0.046]			
**0–120 h**	Mean (SD)	3,326.3 (3,224.6)	3,371.6 (3,084.0)	3,594.3 (3,195.7)	3,998.2 (3,002.8)	3,055.2 (3,318.8)	2,777.7 (3,170.8)
	Diff. vs placebo[a]	−547.0 (−1,217.4, 123.4) [0.110]	−809.1 (−1,481.4, −136.8) [0.018]	−919.4 (−1,591.0, −247.8) [0.007]			
	Diff. vs tramadol[a]	617.0 (69.9, 1164.1) [0.027]	354.9 (−192.3, 902.1) [0.203]	244.7 (−301.4, 790.7) [0.380]			
	Diff. vs celecoxib[a]	−191.0 (−737.4, 355.5) [0.493]	−453.1 (−1,003.7, 97.6) [0.107]	−563.3 (−1,110.4, −16.2) [0.044]			

Between-group differences were analysed using an analysis of covariance model with treatment and qualifying pain intensity at randomization (moderate or severe) as fixed effects, centre as random effect, and pre-dose (0 h) pain intensity as a covariate.

[a]Least-squares mean difference: placebo/tramadol/celecoxib minus CTC 100, 150, or 200 mg (95% confidence interval) [*p* value – two-sided test of no difference for testing null hypothesis that the difference of means is zero].

CTC, co-crystal of tramadol-celecoxib; Diff., Difference; SD, standard deviation.

Table S4 Total pain relief over 0–4, 0–12, 0–24, 0–48, 0–72, 0–96, and 0–120 hours (last observation carried forward, full analysis set)

		CTC 100 mg	CTC 150 mg	CTC 200 mg	Tramadol 100 mg	Celecoxib 100 mg	Placebo
**0–4 h**	Mean (SD) [*n*]	5.69 (3.11) [196]	5.81 (2.95) [200]	6.05 (3.08) [197]	6.01 (3.09) [204]	5.96 (2.95) [190]	4.70 (3.19) [98]
	Diff. vs placebo[a]	−0.85 (−1.53, −0.17) [0.014]	−1.16 (−1.84, −0.48) [<0.001]	−1.26 (−1.95, −0.58) [<0.001]			
	Diff. vs tramadol[a]	0.30 (−0.25, 0.86) [0.282]	−0.00 (−0.56, 0.55) [0.987]	−0.11 (−0.66, 0.44) [0.697]			
	Diff. vs celecoxib[a]	0.32 (−0.24, 0.88) [0.258]	0.02 (−0.55, 0.58) [0.956]	−0.09 (−0.65, 0.47) [0.756]			
**0–12 h**	Mean (SD) [*n*]	19.24 (9.59) [197]	20.51 (9.21) [200]	21.61 (9.94) [198]	21.19 (9.65) [205]	20.68 (9.20) [196]	17.08 (10.35) [102]
	Diff. vs placebo[a]	−1.87 (−3.99, 0.26) [0.085]	−3.66 (−5.79, −1.53) [<0.001]	−4.29 (−6.42, −2.16) [<0.001]			
	Diff. vs tramadol[a]	1.93 (0.19, 3.68) [0.030]	0.14 (−1.61, 1.88) [0.879]	−0.49 (−2.24, 1.25) [0.581]			
	Diff. vs celecoxib[a]	1.73 (−0.03, 3.48) [0.055]	−0.07 (−1.84, 1.70) [0.935]	−0.70 (−2.46, 1.06) [0.436]			
**0–24 h**	Mean (SD) [*n*]	42.61 (20.57) [198]	44.66 (19.41) [200]	46.86 (20.89) [199]	45.95 (19.81) [205]	44.63 (19.91) [197]	37.14 (22.12) [102]
	Diff. vs placebo[a]	−5.01 (−9.52, −0.51) [0.029]	−8.04 (−12.55, −3.52) [<0.001]	−9.17 (−13.68, −4.66) [<0.001]			
	Diff. vs tramadol[a]	3.24 (−0.46, 6.94) [0.086]	0.22 (−3.48, 3.91) [0.908]	−0.91 (−4.61, 2.79) [0.629]			
	Diff. vs celecoxib[a]	2.56 (−1.16, 6.28) [0.177]	−0.46 (−4.21, 3.29) [0.809]	−1.59 (−5.32, 2.14) [0.403]			
**0–48 h**	Mean (SD) [*n*]	91.58 (44.74) [198]	94.61 (41.58) [201]	98.98 (44.74) [199]	97.73 (41.89) [205]	93.11 (42.56) [197]	78.79 (48.78) [102]
	Diff. vs placebo[a]	−12.19 (−21.91, −2.46) [0.014]	−17.06 (−26.81, −7.32) [<0.001]	−19.11 (−28.86, −9.36) [<0.001]			
	Diff. vs tramadol[a]	5.67 (−2.33, 13.67) [0.165]	0.79 (−7.18, 8.76) [0.846]	−1.26 (−9.25, 6.73) [0.758]			
	Diff. vs celecoxib[a]	2.53 (−5.51, 10.57) [0.538]	−2.35 (−10.43, 5.73) [0.568]	−4.40 (−12.45, 3.66) [0.284]			
**0–72 h**	Mean (SD) [*n*]	142.34 (70.15) [198]	146.13 (65.04) [201]	154.51 (69.27) [199]	153.25 (65.14) [205]	144.70 (66.89) [197]	122.35 (75.61) [102]

	Diff. vs placebo[a]	−19.30 (−34.45, −4.15) [0.0013]	−26.30 (−41.48, −11.13) [<0.001]	−30.86 (−46.04, −15.67) [<0.001]			
	Diff. vs tramadol[a]	10.32 (−2.13, 22.78) [0.104]	3.32 (−9.10, 15.73) [0.600]	−1.24 (−13.68, 11.21) [0.845]			
	Diff. vs celecoxib[a]	3.89 (−8.63, 16.41) [0.542]	−3.11 (−15.70, 9.47) [0.627]	−7.67 (−20.21, 4.87) [0.230]			
**0–96 h**	Mean (SD) [*n*]	196.65 (97.07) [198]	201.02 (90.79) [201]	213.35 (95.76) [199]	208.35 (90.09) [205]	199.66 (92.92) [197]	169.37 (104.80) [102]
	Diff. vs placebo[a]	−26.55 (−47.43, −5.67) [0.013]	−35.71 (−56.64, −14.79) [<0.001]	−42.46 (−63.40, −21.53) [<0.001]			
	Diff. vs tramadol[a]	10.83 (−6.34, 28.00) [0.216]	1.66 (−15.45, 18.78) [0.849]	−5.09 (−22.24, 12.07) [0.561]			
	Diff. vs celecoxib[a]	5.41 (−11.85, 22.68) [0.539]	−3.75 (−21.11, 13.61) [0.672]	−10.50 (−27.80, 6.79) [0.234]			
**0–120 h**	Mean (SD) [*n*]	254.83 (126.46) [198]	255.93 (118.30) [201]	275.39 (125.45) [199]	266.20 (118.63) [205]	257.29 (119.62) [197]	219.88 (136.70) [102]
	Diff. vs placebo[a]	−34.37 (−61.43, −7.30) [0.013]	−42.07 (−69.19, −14.95) [0.002]	−53.61 (−80.75, −26.48) [<0.001]			
	Diff. vs tramadol[a]	10.13 (−12.13, 32.39) [0.372]	2.43 (−19.76, 24.61) [0.830]	−9.12 (−31.35, 13.12) [0.421]			
	Diff. vs celecoxib[a]	5.54 (−16.83, 27.92) [0.627]	−2.16 (−24.66, 20.35) [0.851]	−13.70 (−36.12, 8.72) [0.231]			

Between-group differences were analysed using an analysis of covariance model with treatment and qualifying pain intensity at randomization (moderate or severe) as fixed effects, pooled centre as random effect, and pre-dose (0 h) pain intensity as a covariate. Pain relief assessments after first rescue medication were set to missing and imputed by the last available non-missing post-baseline pain relief value.

[a]Least-squares mean difference: placebo/tramadol/celecoxib minus CTC 100, 150, or 200 mg (95% confidence interval) [*p* value – two-sided test of no difference for testing null hypothesis that the difference of means is zero].

CTC, co-crystal of tramadol-celecoxib; Diff., difference; SD, standard deviation.

Table S5 Responder rates, perceptible pain relief and meaningful pain relief (full analysis set)

		**CTC 100 mg (*N* = 207)**	**CTC 150 mg (*N* = 207)**	**CTC 200 mg (*N* = 208)**	**Tramadol 100 mg (*N* = 208)**	**Celecoxib 100 mg (*N* = 206)**	**Placebo (*N* = 102)**
**30% responder rate[a] at 4 h**	*n* (%)	73 (35.3)	81 (39.1)	92 (44.2)	79 (38.0)	70 (34.0)	31 (30.4)
	OR vs placebo[b]	1.21 (0.70, 2.11) [0.488]	1.73 (1.00, 3.00) [0.049]	1.93 (1.12, 3.33) [0.018]			
	OR vs tramadol[b]	0.96 (0.62, 1.48) [0.011]	1.36 (0.89, 2.10) [0.159]	1.52 (0.99, 2.33) [0.055]			
	OR vs celecoxib[b]	0.98 (0.63, 1.52) [0.921]	1.40 (0.90, 2.16) [0.133]	1.56 (1.01, 2.39) [0.044]			
**Time to 30% response,[a] h**	Median (95% CI)	2.02 (1.73, 2.78)	2.22 (1.73, 2.48)	1.73 (1.48, 2.22)	1.98 (1.32, 2.72)	1.78 (1.50, 2.47)	2.73 (1.98, 4.10)
	HR vs placebo[c]	0.96 (0.73, 1.28) [0.804]	1.18 (0.89, 1.55) [0.257]	1.23 (0.93, 1.62) [0.150]			
	HR vs tramadol[c]	0.93 (0.74, 1.16) [0.512]	1.13 (0.91, 1.41) [0.284]	1.18 (0.95, 1.46) [0.140]			
	HR vs celecoxib[c]	0.86 (0.69, 1.08) [0.190]	1.05 (0.84, 1.31) [0.692]	1.09 (0.88, 1.36) [0.433]			
**50% responder rate at 4 h**	*n* (%)	48 (23.2)	51 (24.6)	64 (30.8)	64 (30.8)	49 (23.8)	18 (17.6)
	OR vs placebo[b]	1.31 (0.68, 2.52) [0.423]	1.79 (0.93, 3.43) [0.081]	2.18 (1.15, 4.14) [0.017]			
	OR vs tramadol[b]	0.73 (0.45, 1.18) [0.200]	1.00 (0.62, 1.61) [0.987]	1.22 (0.77, 1.93) [0.409]			
	OR vs celecoxib[b]	0.87 (0.53, 1.43) [0.570]	1.18 (0.72, 1.95) [0.505]	1.44 (0.90, 2.33) [0.132]			
**Time to 50% response,[a] h**	Median (95% CI)	8.45 (3.73, 17.95)	11.95 (5.93, 18.15)	3.98 (3.00, 7.93)	4.00 (2.73, 11.95)	5.97 (3.48, 12.95)	23.93 (5.93, 36.03)
	HR vs placebo[c]	1.03 (0.76, 1.39) [0.852]	1.16 (0.86, 1.56) [0.337]	1.31 (0.97, 1.75) [0.074]			
	HR vs tramadol[c]	0.91 (0.71, 1.15) [0.414]	1.02 (0.80, 1.29) [0.888]	1.15 (0.92, 1.44) [0.233]			
	HR vs celecoxib[c]	0.93 (0.73, 1.18) [0.547]	1.04 (0.82, 1.33) [0.729]	1.18 (0.94, 1.49) [0.165]			
**Time to perceptible pain relief, h**	Median (95% CI)	2.48 (2.02, 2.98)	2.73 (2.02, 3.08)	2.32 (1.98, 3.73)	2.97 (1.92, 3.78)	2.98 (2.25, 4.08)	2.50 (2.03, 4.02)
	HR vs placebo[c]	1.08 (0.81, 1.43) [0.606]	1.16 (0.88, 1.54) [0.284]	1.03 (0.78, 1.36) [0.839]			

	HR vs tramadol[c]	1.17 (0.92, 1.48) [0.194]	1.26 (1.00, 1.59) [0.049]	1.12 (0.88, 1.41) [0.355]			
	HR vs celecoxib[c]	0.99 (0.79, 1.25) [0.944]	1.07 (0.85, 1.35) [0.551]	0.95 (0.75, 1.19) [0.648]			
**Time to meaningful pain relief, h**	Median (95% CI)	7.93 (6.02, 12.00)	12.00 (6.05, 12.08)	6.00 (4.28, 7.87)	7.92 (6.00, 11.93)	6.12 (6.00, 12.02)	29.93 (8.10, 36.12)
	HR vs placebo[c]	1.40 (1.02, 1.92) [0.038]	1.39 (1.01, 1.91) [0.041]	1.52 (1.11, 2.08) [0.009]			
	HR vs tramadol[c]	0.93 (0.73, 1.19) [0.583]	0.93 (0.73, 1.18) [0.544]	1.02 (0.80, 1.29) [0.891]			
	HR vs celecoxib[c]	1.03 (0.80, 1.32) [0.821]	1.02 (0.80, 1.31) [0.859]	1.12 (0.88, 1.43) [0.361]			

[a]30% or 50% reduction from baseline in pain intensity–visual analogue scale (if a patient was missing a timepoint or took rescue medication prior to a timepoint, they were considered to be a non-responder).

[b]OR: CTC 100, 150, or 200 mg versus placebo/tramadol/celecoxib (95% CI) [*p* value – two-sided test of no difference for testing null hypothesis that the OR is 1]. These data were analysed using a logistic regression model with treatment and QPI at randomization (moderate or severe) as fixed effects, centre as a random effect, and pre-dose (0 h) pain intensity as a covariate.

[c]HR: CTC 100, 150, or 200 mg versus placebo/tramadol/celecoxib (95% CI) [*p* value – two-sided test of no difference for testing null hypothesis that the HR is 1]. These data were analysed using a Cox proportional hazard model with treatment and QPI at randomization (moderate or severe) as fixed effects, centre as a random effect, and pre-dose (0 h) pain intensity as a covariate.

CI, confidence interval; CTC, co-crystal of tramadol-celecoxib; HR, hazard ratio; *n*, number of patients with data available; *N*, number of patients in analysis set; LOCF, last observation carried forward; OR, odds ratio; QPI, qualifying pain intensity.

Table S6 Use of rescue medication (full analysis set)

		CTC 100 mg (*n* = 207)	CTC 150 mg (*n* = 207)	CTC 200 mg (*n* = 208)	Tramadol 100 mg (*n* = 208)	Celecoxib 100 mg (*n* = 206)	Placebo (*n* = 102)
**Average dose of rescue medication**	Dose per 24 h, mg; mean (SD)	197.4 (430.3)	186.9 (444.2)	165.7 (384.0)	140.6 (332.3)	290.9 (539.1)	274.5 (506.5)
	Diff. vs placebo[a]	76.9 (−18.7, 172.6) [0.115]	92.1 (−3.8, 188.0) [0.060]	117.2 (21.4, 213.1) [0.017]			
	Diff. vs tramadol[a]	−53.2 (−131.3, 24.8) [0.181]	38.0 (−116.1, 40.0) [0.339]	12.9 (−90.8, 65.0) [0.745]			
	Diff. vs celecoxib[a]	82.7 (4.8, 160.7) [0.038]	97.9 (19.4, 176.5) [0.015]	123.0 (45.0, 201.1) [0.002]			
**Time to 1st dose of rescue medication**	Time for 1st quartile of patients, h (95% CI)	7.00 (3.30, 11.17)	10.45 (7.33, 13.98)	10.48 (7.35, 24.50)	13.92 (4.38, 47.92)	6.53 (3.50, 10.48)	3.60 (2.20, 11.45)
	HR vs placebo[b]	0.83 (0.57, 1.21) [0.333]	0.73 (0.50, 1.06) [0.098]	0.67 (0.46, 0.98) [0.038]			
	HR vs tramadol[b]	1.32 (0.95, 1.83) [0.099]	1.16 (0.83, 1.61) [0.390]	1.06 (0.76, 1.49) [0.734]			
	HR vs celecoxib[b]	0.90 (0.66, 1.22) [0.487]	0.79 (0.58, 1.07) [0.125]	0.72 (0.53, 0.99) [0.041]			

[a]Least-squares mean difference: placebo/tramadol/celecoxib minus CTC 100, 150, or 200 mg (95% CI) [*p* value – two-sided test of no difference for testing null hypothesis that the difference of means is zero]. These data were analysed using an analysis of covariance model with treatment and QPI at randomization (moderate or severe) as fixed effects, centre as random effect, and pre-dose (0 h) pain intensity as a covariate.

[b]HR: CTC 100, 150, or 200 mg versus placebo/tramadol/celecoxib (95% CI) [*p* value – two-sided test of no difference for testing null hypothesis that the HR is 1]. These data were analysed using a Cox proportional hazard model with treatment and QPI at randomization (moderate or severe) as fixed effects, centre as random effect, and pre-dose (0 h) pain intensity as a covariate.

CI, confidence interval; CTC, co-crystal of tramadol-celecoxib; Diff., difference; HR, hazard ratio; QPI, qualifying pain intensity; SD, standard deviation.

Fig. S1 Study design. AE, adverse event; BID, twice daily; CTC, co-crystal of tramadol-celecoxib; FU, follow-up; QID, four times daily; R, randomization; V, Visit. The telephone icon indicates that the visit could be conducted by telephone. aV5 and V6 were performed at the study site for patients participating in pharmacokinetic sampling.

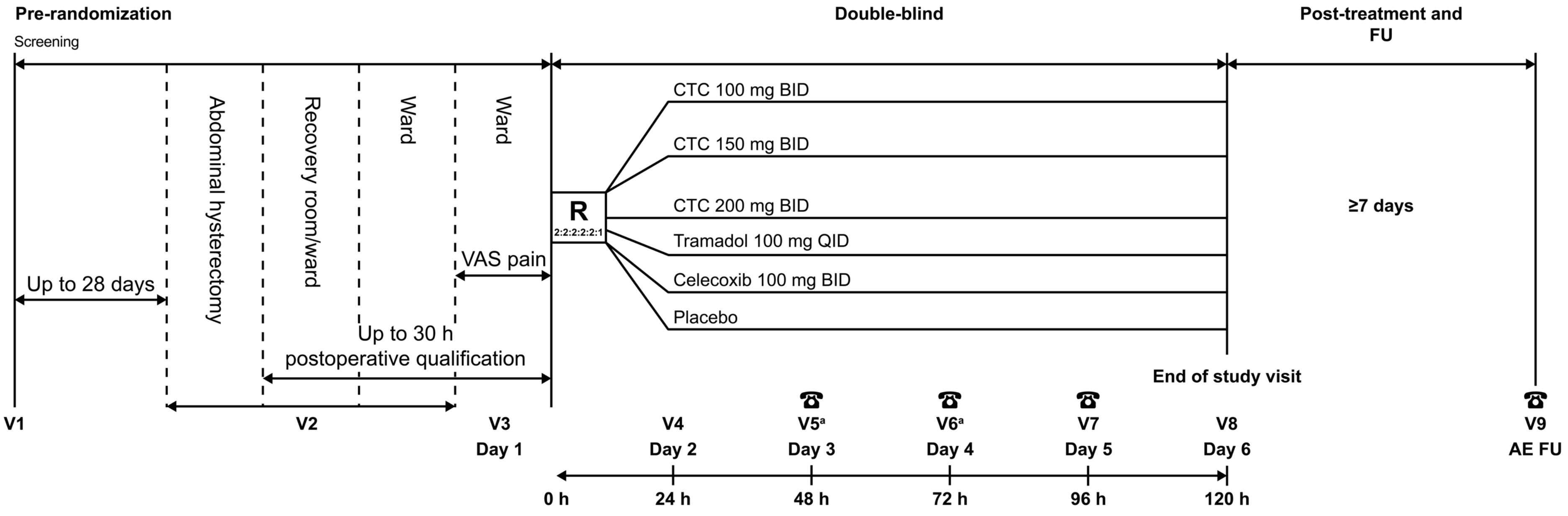

.

Fig. S2 Patient disposition. AE, adverse event; CTC, co-crystal of tramadol-celecoxib

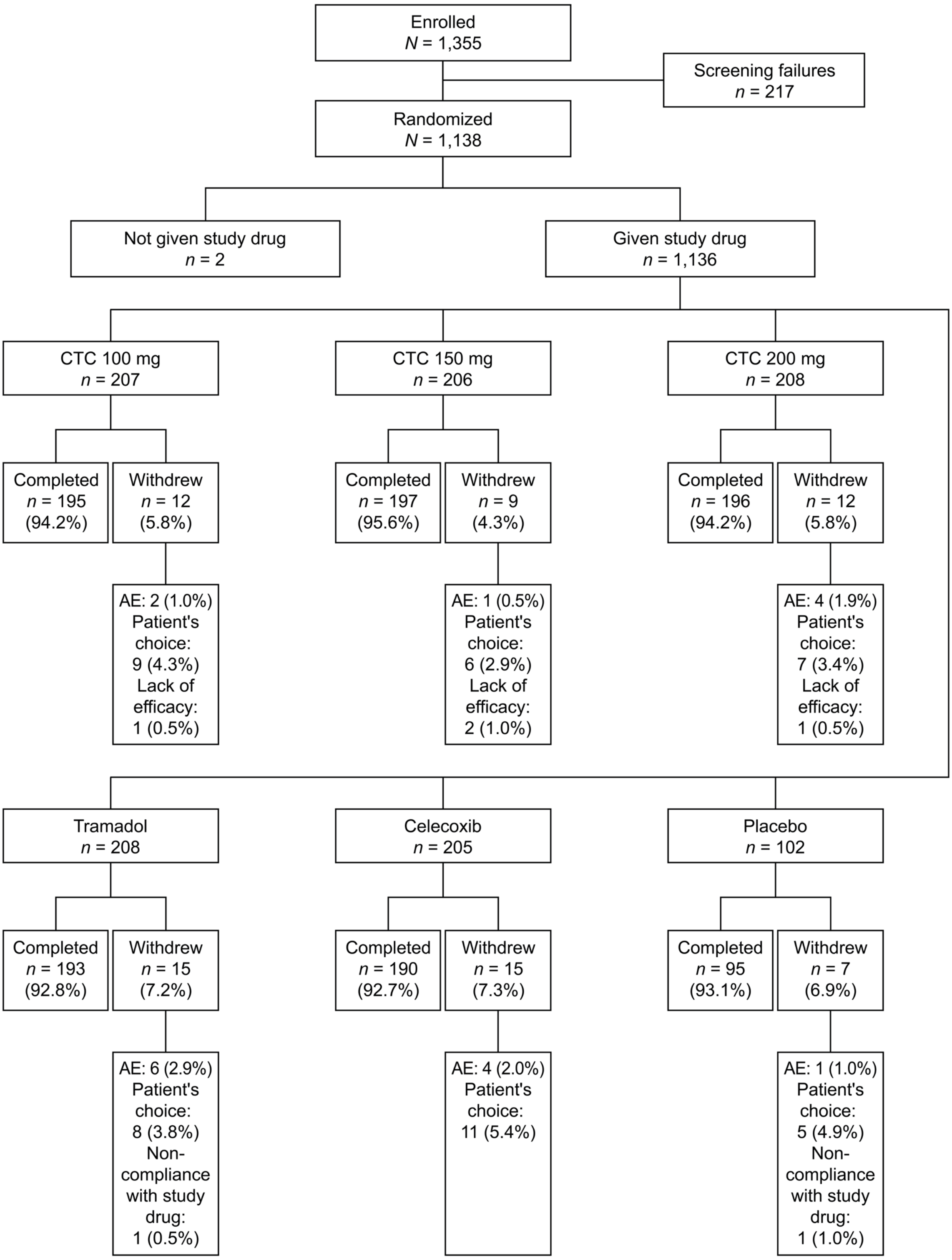

Fig. S3 Summary of (a) TEAEs and study-drug-related TEAEs and (b) the most frequently occurring TEAEs (safety analysis set). ALT, alanine aminotransferase; AST, aspartate aminotransferase; CTC, co-crystal of tramadol-celecoxib; GGT, gamma-glutamyl transferase; TEAE, treatment-emergent adverse event

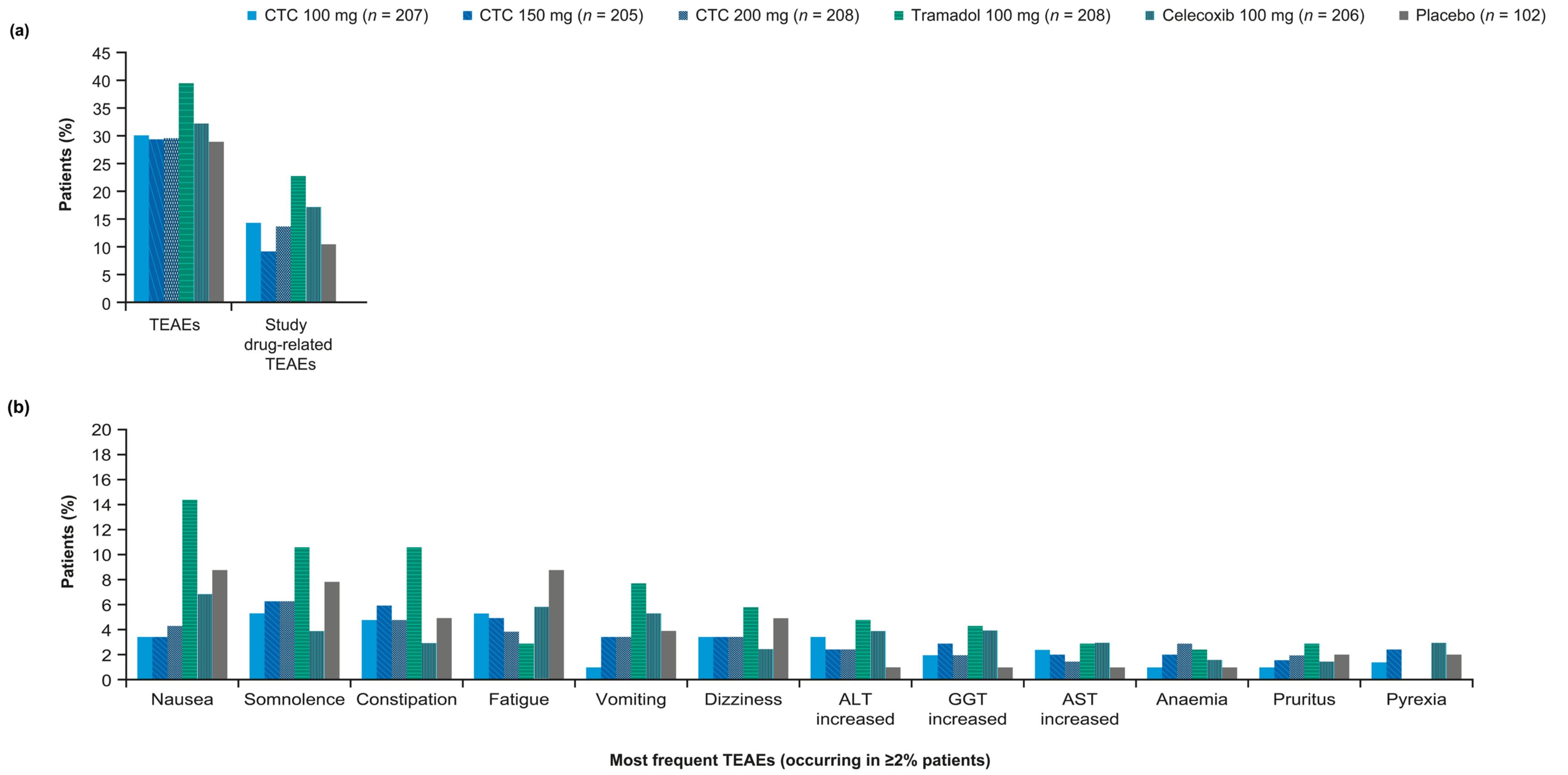

Fig. S4. Plasma drug levels (PK analysis set): (a) tramadol plasma concentration data (mean ± SD), (b) O-desmethyltramadol plasma concentration data (mean ± SD) and (c) celecoxib plasma concentration data (mean ± SD). CTC, co-crystal of tramadol-celecoxib; SD, standard deviation.

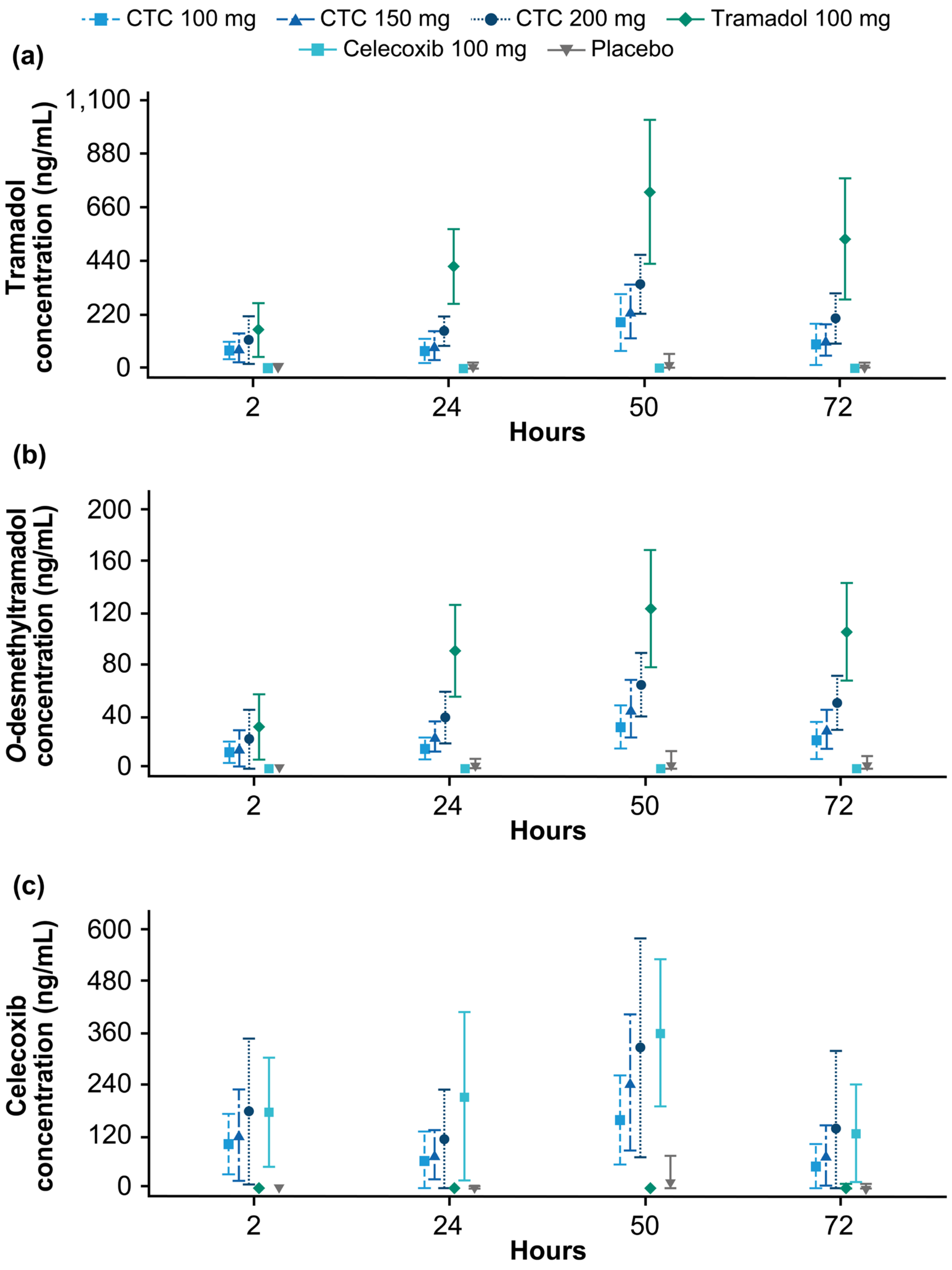

Supplement: Supplementary file 1 — Appendix S1 [file EJP-26-2083-s001.pdf]
